# The health knowledge mechanism: evidence on the link between education and health lifestyle in the Philippines

**DOI:** 10.1007/s10198-017-0950-2

**Published:** 2018-01-03

**Authors:** Roman Hoffmann, Sebastian Uljas Lutz

**Affiliations:** 1 Wittgenstein Centre for Demography and Global Human Capital (IIASA, VID/ÖAW, WU), Vienna Institute of Demography, Austrian Academy of Sciences, Welthandelsplatz 2, Level 2, 1020 Vienna, Austria; 2 Institute for Macroeconomics, Vienna University of Economics and Business, Welthandelsplatz 1, 1020 Vienna, Austria

**Keywords:** Education, Health knowledge, Health lifestyle, Allocative efficiency, Developing country, Philippines, D83, I12, I14, I15, I26, O2

## Abstract

Studies have found substantial differences in health-related behavior and health care usage between educational groups, which may explain part of the well-documented educational gradient in health. The allocative efficiency hypothesis offers a behavioral explanation for these reported differences. According to this theory, the educated possess more health knowledge and information, allowing them to make better health choices. We perform a mediation analysis to study this mechanism using original survey data from the Philippines, a lower-middle-income country. As an extension of previous empirical research, we construct a comprehensive index that captures different dimensions of health knowledge. Using generalized propensity scores, we find strong support for the allocative efficiency argument. Schooling is significantly associated with health knowledge levels, which explain up to 69% of the education effect on health lifestyle. This corresponds to twice the mediation strength of economic resources, suggesting an important role of this factor in explaining education effects on health decisions.

**Electronic supplementary material:**

The online version of this article (10.1007/s10198-017-0950-2) contains supplementary material, which is available to authorized users.

## Introduction

Previous studies have documented considerable inequalities in health between educational groups. Better-educated people have a lower mortality, experience less often harmful diseases, and feel overall healthier than their less-educated peers [26]. One behaviorally based explanation for educational differentials in health is provided by the allocative efficiency hypothesis, which extends on the reasoning of early health capital models [27, 51, 63]. The theory suggests that education raises a person’s health knowledge, allowing the educated to choose a more efficient input mix in the health production process, i.e., to make better health decisions, leading to improved health outcomes [41, 57].

In line with this argument, previous studies have found education to positively influence health-related lifestyles and behaviors, such as smoking [36, 38, 40, 64], drinking [36], or care seeking [53, 56]. Despite this evidence, little is known about the actual mechanisms through which education affects behavioral patterns. The few studies that test for the relevance of knowledge as a mediating channel in explaining education effects on health decisions come to mixed results. While most of them do at least partially confirm the allocative efficiency predictions, in none of them health knowledge accounts for a considerably large share of education effects [14, 41, 48, 49]. The evidence suggests that other mechanisms, such as differences in productive efficiency or omitted third factors, might drive the effects.

Unlike previous studies, which rely predominantly on data from developed countries, we provide a test of the allocative efficiency hypothesis in the Philippines, a lower-middle-income country. In this setting, access to health-related information may be restricted, raising the importance of educational institutions in improving knowledge and disseminating information about disease threats and appropriate health practices [17]. As a further contribution to the literature, we try to overcome a major challenge in the testing of the allocative efficiency mechanism, which is the measurement of respondents’ health knowledge. In previous studies, indicators tended to measure knowledge in a restricted and simplistic way, limiting attention to knowledge about one health topic, such as the risks of smoking, which might not well capture the multi-dimensionality and complexity of the construct [3, 14, 41, 48]. Using self-collected data, we construct an extensive knowledge index, which is tailored to the local context. The measure is based on 28 openly answerable questions about various health topics, such as disease prevention, medical treatments, child health, family planning, and sexual transmittable diseases. Using this novel measure, we find knowledge to play a pivotal role in explaining education effects, lending strong support to the allocative efficiency argument.

Previous research considered mostly individual behavioral indicators [36, 53, 64]. Although commonly used, this approach does not account for the fact that health decisions are not made in isolation, but may depend on each other in the health production function. For instance, an individual could try to compensate a bad health behavior, such as the consumption of unhealthy food, with a good health behavior, such as regular exercising. We consider an individual’s health lifestyle, which we define as an aggregate expression of different health practices, such as disease prevention, eating habits, and exercising. Based on 14 binary coded behaviors, respondents are categorized as having either an overall healthy or unhealthy lifestyle using techniques of cluster and latent class analysis [29, 39]. To test for the consistency of our results, we perform the analysis not only for the aggregate lifestyle measures but also separately for the single behavioral indicators.

We estimate respondents’ propensity to get educated based on their pre-education characteristics, such as socio-economic status in childhood, parental educational background, and birth region characteristics. The resulting propensity scores are used as an aggregate control variable in our analysis to control for potentially confounding background variables that may influence both the selection into education and the health lifestyle. A special feature of our analysis is the estimation of generalized propensity scores (GPS) for a continuous treatment variable, years of education, as developed by Hirano and Imbens [32]. In addition to logit models, we use the KHB method in our mediation analysis. This procedure allows us to decompose direct and indirect (mediated through knowledge) education effects across non-linear nested models with binary outcome variables [11, 43]. Following previous research [14, 25], we control for other theoretically relevant mediating factors, such as wealth and risk preferences, in additional models to test for the robustness of our findings and to identify the isolated effect of knowledge on health lifestyle. The data for this study was collected among the female heads of 1064 low-income households in the greater area of Metro Manila, the capital of the Philippines. All of our respondents were clients of a social development microfinance institution at the time of the survey.

Controlling for the individual propensity to get educated, we find strong support for the predictions of the allocative efficiency hypothesis. Education is strongly related to health knowledge levels, which in turn are positively associated with health lifestyle. The effect sizes are substantial: An additional year of schooling raises the probability of having a healthy lifestyle by about 3.5%. Once knowledge as a mediating factor is introduced in the full models, education effects are significantly reduced by between 65 and 69% depending on the lifestyle typologisation used (cluster vs. latent class analysis, respectively). As a benchmark, wealth as a measure of economic resources explains up to 32% of the observed education effects, further highlighting the role of knowledge and information as an important mediating channel. The results also hold if we perform the analysis separately for each health behavior, even though we observe some heterogeneity in effect sizes and mediation strength across the different indicators. Interestingly, knowledge has a positive influence also for some of the health behaviors for which education does not seem to matter, suggesting that the variable can also be of importance independent of a person’s level of education.

Although our empirical results are in line with the predictions of the allocative efficiency argument, it is worth noting that our study faces some limitations. Due to the cross-sectional nature of our data we cannot make causal claims. Even though we attempt to control for the pre-education background of our respondents, our key explanatory variables education and knowledge may still be potentially endogenous. As an explorative mediation analysis, the strength of our study does not lie in the rigorous causal identification, but in the detailed examination of the knowledge mechanism as a possible explanation for education effects. Our findings can serve as an indirect test of the allocative efficiency hypothesis and as indication for the importance of knowledge as mediator in explaining health-related outcomes in a developing country context.

The remainder of the paper is structured as follows. The next section introduces the theoretical framework of our analysis and summarizes previous empirical evidence. The following section gives an overview of the data, the identification strategy, and the operationalization of the key variables. The next section presents the results and the next section discusses the findings against the background of the current literature. The final section concludes.

## Theoretical framework and empirical literature

There is strong, although not uncontested, evidence for a positive causal link between education and health (see [20] for an overview). The seminal Grossman [27] model laid the theoretical foundation to which most of the present empirical and theoretical work refers to. Inspired by classical human capital models [7, 8] Grossman considers health as both an investment and consumption good. In his model, he directly addresses the demand for health as a durable capital stock. He argues that an individual’s level of education enhances the productive capacity for market employment as well as commodity production. Accordingly, better-educated people are able to reach better health states at lower costs, because education renders the individual’s health production process more efficient [28, 30, 35]. Here productive efficiency refers to a greater outcome keeping the input allocation constant.

**Fig. 1:**
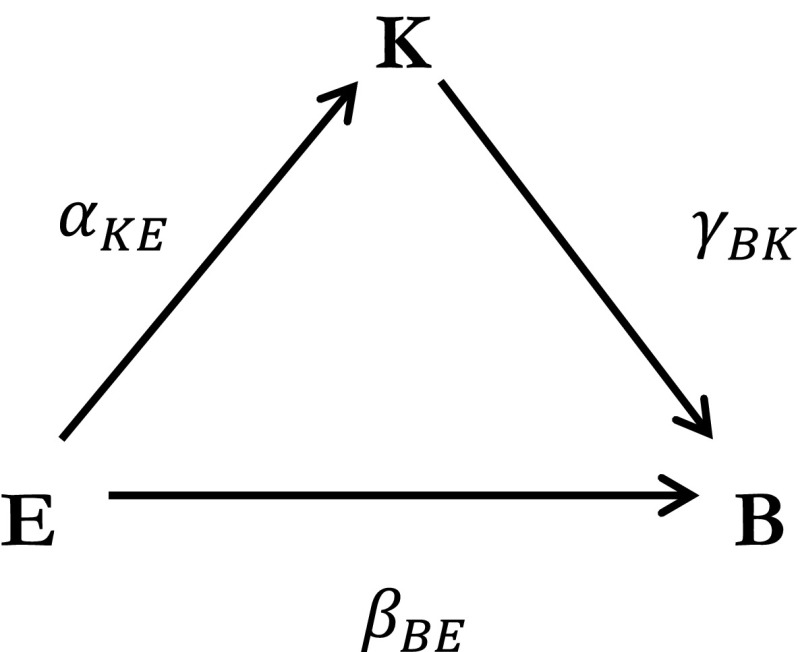
The allocative efficiency mechanism

A complementary theoretical explanation is the allocative efficiency hypothesis [3, 41, 57]. In this theory, schooling improves an individual’s knowledge about health and the implications of her health choices, leading to a superior allocation of inputs in the health production process [47]. This implies that persons with different degrees of education and knowledge choose different health inputs, i.e., they show different health behaviors. Schooling can raise health knowledge either directly, for example in school delivered health trainings, or indirectly by raising a person’s absorptive capacity for health information, in particular if it is complex [59]. In line with this argument, [65] finds that the more educated show a stronger and faster reaction to an HIV information campaign in Uganda. Similar results are presented by [2] for behavioral responses to novel information about the impacts of smoking or [45] for the adoption of preventive behaviors as a reaction to varying personal cancer risks. The main theoretical mechanism of the allocative efficiency hypothesis is depicted in Fig. 1.

We derive three testable predictions based on the allocative efficiency hypothesis, which claims that behavior B is a function of education E and health knowledge K with $B = f(E, K\left( E \right))$. First, education E is predicted to positively influence knowledge K (H_1_: $\alpha_{KE } = \delta K/\delta E > 0$); second, the total (direct + indirect) effect of education on health behavior B is expected to be positive (H_2_: $\beta_{BE} = {\text{d}}B/{\text{d}}E = \delta B/\delta E + (\delta B/\delta K) \cdot (\delta K/\delta E) > 0$); and third, knowledge K is predicted to be one of the mechanisms explaining the relationship between education and behavior as a mediating factor meaning that an indirect effect through the knowledge channel exists (H_3_: ${\text{d}}B/{\text{d}}E - \delta B/\delta E = (\delta B/\delta K) \cdot (\delta K/\delta E) > 0)$.

In line with the second prediction, various studies report positive education effects on health behaviors [4, 10, 40, 50, 61]. For instance, using data on a major schooling information campaign in the Dominican Republic, [36] find that schooling significantly reduces smoking and delays the onset of daily or regular drinking among male students. On the other hand, only few studies have directly tested for the mediating effect of knowledge on the education–health lifestyle relationship.^1^ In one of the first studies, [41] shows that health knowledge explains part of the positive effect of education on health-related behaviors, such as smoking, drinking, or exercising. However, even under control for health knowledge, a large share of the education effect remains unexplained, suggesting that schooling may additionally have an impact on behavior through channels other than knowledge. Similarly, [14] find only a modest impact of knowledge on the education gradient in health behavior, using two measures of knowledge about the risks of smoking and drinking. They report a decrease of 17% of education effects on current smoking and no change in the coefficients for drinking once the knowledge measure is introduced in the model (See also [48]). Unlike the other studies, [49] do not find significant changes in education effects on smoking and drinking after controlling for knowledge. Also, [52], who uses a proxy for knowledge about the risks of HIV in Sub-Saharan Africa, does not find that regions with more health knowledge show less-risky sexual behavior.

Health knowledge seems to play a role, but does not appear to be the main explanatory factor in the education–health lifestyle relationship. The question remains if it is actually education that raises knowledge, or if another factor, such as parental background, is driving the effects. Few studies have tried to identify the direct effect of education on health knowledge $\alpha_{KE }$, which can be understood as a pre-requisite for the allocative efficiency argument to hold. [3] use two waves of the US National Longitudinal Survey of Youth. The data contains information about individuals who were aged 12–16 in the initial survey period in 1996. In their identification the authors use the exogenous variations in the timing of the follow-up surveys in 2002 (from 54 to 78 months after the initial interviews) as instruments for school attendance. They find only weak evidence for education effects on knowledge, concluding that “allocative efficiency is not likely to be the main reason for why education improves health” (p. 811). Similarly, [37], who use data of the UK Health and Lifestyle Survey and compulsory schooling reforms in 1947, do not find education to have a positive effect on knowledge, further challenging the validity of the allocative efficiency argument.

## Research design and methods

### Data

In this study, we consider education effects on health lifestyle in a developing country context, where health behaviors and its determinants may strongly differ from those in developed countries, which have been the focus of most previous research. We use cross-sectional data collected by the authors in April 2015 in the Philippines (see supplement S1: Sample selection and data collection). The sample of respondents was randomly drawn among the female clients of a social development microfinance institution, the Kasagana-Ka Development Center Inc. (KDCI), using a multi-stage sampling procedure. In total, 1064 KDCI clients from 70 different microfinance groups in Metro Manila and the surrounding province of Rizal were interviewed with an 18 page long standardized questionnaire.

As complementary data we collected geographical information on the locations of respondents’ homes and public health facilities, such as hospitals or primary health care units, in the study areas. Furthermore, we derive information about the birth province of our respondents using data from the Philippine Census of Population and Housing for the years 1948, 1960, 1970, 1975, 1980, and 1990. The data, which was provided to us by the Philippine Statistical Authority and encoded by the authors, was merged with respondent’s individual data to obtain information about the environmental conditions in the respective province at the time of respondent’s birth.

### Empirical strategy

We follow a three-step estimation strategy, which is outlined in more detail in section S2 in the supplementary material. First, we estimate respondent’s propensity to obtain a specific education level with years of schooling as continuous variable of interest [32]. The generalized propensity score (GPS) derived from this first step is then used in the subsequent analyses as an aggregate control variable to efficiently control for the pre-education background of respondents. For the propensity score estimation, we use information on relevant personal pre-education characteristics: Parental education and literacy, absence of parents in childhood, short-term memorability as a proxy for fluid cognitive abilities, early work experience as a proxy for wealth in childhood, and age in four categories (< 35, 35–44, 45–54, > 54). Besides personal characteristics, we include different potentially relevant birth province characteristics into the models. Here, we exploit the particularly rich variation in provinces of origin in our sample. Our respondents came from 68 out of 81 provinces and only 25% were born in one of the study areas in Metro Manila or Rizal Province. In particular, we use context variables, which capture the economic development level and educational infrastructure in the province and are expected to influence individual education decisions, namely, the distance of the birth province to the capital, the provincial literacy rate, the population density, the elementary school completion rate, and the electrification rate in the province. All variables, except distance to the capital, were dichotomized at the median to improve the balancing of the sample.

In the second step, we estimate the dose response effect of education E measured in years of schooling on health knowledge K while controlling for the GPS. This procedure is repeated in the third step, in which we are interested in the effect of education on respondent’s health lifestyle and behaviors. Methods of cluster analysis and latent class modeling are used for the lifestyle typologisation which is based on 14 selected health behaviors [29, 39]. As all behavioral outcomes are binary coded, we use logit models in the estimation. Education effects are estimated first without (baseline model) and then under control for knowledge K as a potential mediating factor (extended model). The change in the education coefficients serves as indication for the explanatory power of knowledge in the education–health lifestyle relationship. For our mediation analysis we employ the KHB method, which allows for the comparison of coefficients across non-linear nested models and provides an estimate for the strength of mediation for the additionally included factor [11, 33, 43].

1 $$P\left( {B = 1|E, {\text{GPS}}, C} \right) = \frac{{\exp (\beta_{0} + E\beta_{1} + {\text{GPS}}\beta_{2} )}}{{1 + \exp (\beta_{0} + E\beta_{1} + {\text{GPS}}\beta_{2} )}}$$

2 $$P\left( {B = 1|E, {\text{GPS}}, C,K} \right) = \frac{{\exp (\gamma_{0} + E\gamma_{1} + {\text{GPS}}\gamma_{2} + K\gamma_{3} )}}{{1 + \exp (\gamma_{0} + E\gamma_{1} + {\text{GPS}}\gamma_{2} + K\gamma_{3} )}}$$

The main analysis above is repeated in a final step in which we additionally control for a set of other factors M in the baseline model [1], which have been proposed as alternative mediating channels in the literature [14, 21, 25, 54]. Like before, we add knowledge K to the right-hand side of the equation to test for changes in the education coefficient. By controlling for the additional variables M, we can ensure that any change in the education effect is due to the inclusion of knowledge and not another omitted mediating factor. We include measures for economic resources, risk preferences, access to health facilities, social capital, marital status, number of children in the household, religiosity, and the subjective health status. Furthermore, we control for the education and knowledge background in the entire microfinance network and in the direct peer group to take potential contextual peer effects into consideration [19, 34]. These have been shown to be of importance for health behaviors in different other settings [23]. Detailed information on the construction of both the pre-education background variables and the mediating variables used as additional controls can be found in the supplementary material (S3: Measurement of pre-education characteristics and S4: Measurement of additional mediating factors).

### Measurement of health knowledge

The health knowledge measure is based on 28 items, a considerably larger number than in other studies. Using a more extensive set reduces the weight of the single items, which increases the overall reliability of the measure. Furthermore, the instrument differs in terms of scope from previously used measures: The single items are not restricted to specific aspects of knowledge, but represent a broad range of context-relevant health topics, such as disease care or reproductive health. With this we attempt to better capture the multidimensionality and complexity of the construct, acknowledging that knowledge may be difficult to assess with simple indicators.^2^ To further increase the validity of the instrument, we do not present respondents with pre-defined answer categories to avoid anchoring effects and the use of simple guessing strategies, such as replying yes to all questions [14]. Instead, respondents were allowed to give open answers in order to gain a more accurate and exhaustive picture of a person’s health knowledge.

Table 6 in the appendix presents the single items included in the health knowledge index and provides summary statistics. All answers were categorized and evaluated based on minimum answer rules. Taking the non-medical background of the survey respondents into account, some answer rules are rather simple and only control for respondent’s basic understanding of a method or disease.^3^ If an open question required several correct answers, the answer rule represents a minimum number of correct answers. This number was defined based on a median split, such that the item was classified as correct for not more than 50% of the sample.

**Fig. 2:**
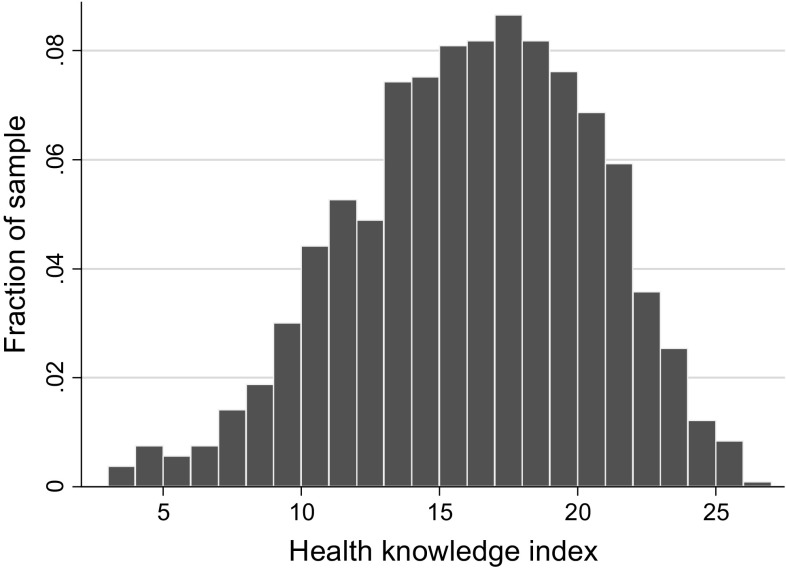
Distribution of health knowledge index

The single items were additively combined in a health knowledge index (Cronbach’s alpha: 0.769). The index has a scale from 0 to 28 and an approximate bell-shaped distribution around the mean of 15.7 (see Fig. 2). Our findings are not sensitive to the use of different aggregation techniques, such as (i) an additive index of the normalized knowledge items *k* where each item $k_{j} \in \left\{ {0\left. {,1} \right\}} \right.$ was transformed by subtracting the mean of correct answers of all respondents and dividing it by the standard deviation, or (ii) an index for which single items were weighted based on principal component analysis (see supplement S6: Sensitivity tests).

While we focus in our analysis on the role of general health knowledge, we also perform various robustness checks in which we test whether respondents with specific health knowledge, e.g., about strategies to prevent hypertension, are more likely to undertake specific beneficial behaviors, such as exercising or healthy food consumption (see supplement S6: Sensitivity tests).

### Measurement of health lifestyle

We derive a health lifestyle typology (healthy vs. unhealthy lifestyle) based on 14 different behavioral indicators, which are all highly relevant for the low-income context of our sample and the health challenges faced in the Philippines. Several of the indicators, e.g., on exercising or nutrition, have been adopted from previous studies [14, 53]. All indicators were dichotomized for the analysis and take the value one if the respondent pursued a certain behavior that is (presumably) beneficial for her health. In the following paragraph, the values in parentheses indicate the share of respondents who showed a specific behavior in our sample. Table 7 in the appendix gives more detailed information about the operationalization of the single indicators.

The indicators measure if the respondent was exercising regularly (25.4%), consuming fruits regularly (48.4%), able to keep a diet (65.9%), underwent a routine check-up in the last year (44.5%), planned to undergo a routine check-up in the next year (64.9%), had a personal professional health care provider (61.9%), made use of breast self-examination in the past 3 months (32.5%), ever used family planning methods (83.8%), regularly washed her hands throughout the day (54.8%), did not drink any untreated/unfiltered water in the past 3 days (47.4%), was insured with the Philippine public health insurance Phil Health (20.9%), actively sought general health-related information in the past 3 months (52.3%), learned new information about health threats in the past 6 months (41.4%), and searched for information about family planning in the past 12 months (18.0%).

Based on these indicators respondents were categorized as having either an overall healthy or unhealthy lifestyle using cluster and latent class analysis [29, 39]. As exploratory data mining techniques, which have been used in the previous lifestyle literature [15, 46, 58, 60], both methods identify homogenous groups of cases taking into account potential interdependencies between the considered health practices. Transforming the multiple indicators into a unified dichotomous lifestyle measure enables us to compare the estimates for the aggregate lifestyle outcome with the estimates for the separate health behaviors, which are all binary coded. We can hence employ a consistent and coherent estimation framework using logit models and the KHB method to analyze education effects and changes in effect sizes. We also created lifestyle outcomes with multiple categories using the same methodology, but the chosen binary outcome resulted in the greatest fit with the data.

Table 1 informs us about the percentage of people showing a certain health behavior by lifestyle category. As can be inferred from the table, the dichotomous lifestyle variables effectively split the sample along the multiple dimensions revealing clear differences in health practices between respondents with an overall healthy and those with an unhealthy lifestyle. This suggests that the derived lifestyle typologisation is informative.

**Table 1:** Summary statistics for different lifestyle typologies

	Unhealthy lifestyle		Healthy lifestyle		Difference	
	Cluster	Latent	Cluster	Latent	Cluster	Latent
Exercising	0.17	0.16	0.36	0.38	0.19***	0.22***
Fruit consumption	0.33	0.34	0.70	0.67	0.37***	0.33***
Keeping a diet	0.64	0.63	0.69	0.70	0.05	0.07**
Routine check-up last year	0.25	0.28	0.72	0.67	0.48***	0.39***
Routine check-up next year	0.53	0.52	0.81	0.82	0.28***	0.30***
Healthcare provider	0.50	0.48	0.79	0.81	0.29***	0.32***
Breast self-examination	0.16	0.15	0.55	0.57	0.39***	0.42***
Ever used family planning	0.8	0.78	0.89	0.91	0.09***	0.13***
Hand-washing	0.47	0.48	0.67	0.64	0.20***	0.16***
Not drinking untreated water	0.42	0.44	0.55	0.53	0.13***	0.09***
Public health insurance	0.14	0.11	0.31	0.34	0.17***	0.23***
Search for health information	0.33	0.32	0.79	0.79	0.46***	0.47***
Learning info about diseases	0.27	0.28	0.62	0.60	0.35***	0.32***
Search info about family planning	0.14	0.12	0.24	0.27	0.10***	0.15***
Observations	612	612	442	452	1054	1064^a^
Percent of total	58.2	57.5	41.8	42.5	100	100

Lifestyle typologies based on cluster and latent class analysis. All health behavior indicators are binary coded. *Z* tests are used to test for mean differences between the groups

* *p* ≤ 0.1, ** *p* ≤ 0.05, *** *p* ≤ 0.01

^a^ Note that while cluster analysis is sensitive to missing values for the single behavioral indicators, this is not the case for latent class analysis, explaining the differences in sample sizes

As an extension, we re-estimated our main models using two continuous lifestyle outcomes instead of the binary ones, allowing for greater precision in the estimation and to test for the robustness of our findings. The first continuous outcome was created by summing up the different health behavior indicators directly (additive index). For the second continuous outcome, the indicators were weighted using principal component analysis (weighted index). We used OLS to estimate the education and knowledge effects. To make the coefficients comparable to the logit models, we normalized both continuous outcomes to a range from 0 to 1.

## Results

### Propensity score estimation

While we mainly focus on the estimation of education effects on knowledge and health lifestyle as central outcomes, we provide here a brief overview of the estimation of the individual propensity scores, which are used to efficiently control for respondent’s pre-education characteristics in the subsequent models. In our estimation, we closely follow the procedures outlined in [32, 42]. Table 8 in the appendix shows the results of the propensity score estimation. Although the model explains a significant share of the variation in schooling, it is not able to exhaustively capture the selection into education challenging a causal interpretation of the estimated effects. A more lengthy discussion of the results including tests of the central assumptions is provided in the supplementary material (S5: GPS estimation and test of assumptions).

Among the pre-education characteristics, we find that parental education level and literacy exert a particularly strong effect on children’s education. At the same time, growing up in an economically more fortunate environment and having higher cognitive abilities are associated with higher educational achievements. Among the provincial background characteristics, we find that respondents from provinces with a below median elementary school completion rate had a significantly lower education level, which might be due to differences in schooling infrastructures across the provinces. We do not find statistically significant effects for any of the other provincial background variables.

### Education effect on health knowledge

Table 2 shows the results of the GPS-adjusted estimation of education effects on health knowledge. The results clearly confirm the first allocative efficiency prediction. An additional year of schooling is estimated to significantly raise health knowledge by about 0.534 points on the knowledge index. This represents an increase of the knowledge index of 1.9% with every school year (or a substantial 0.35 standard deviation increase with a 1 standard deviation increase in education).

**Table 2:** Linear dose response estimation: effect of education on health knowledge

	Knowledge
Years of education	0.534*** [0.048]
GPS	0.255 [3.281]
Neighborhood 2	1.177*** [0.344]
Neighborhood 3	1.781*** [0.339]
Constant	9.604*** [0.606]
*N*	1041
Adj. *R*^2^	0.143
AIC	5878.9

Coefficients in cells, standard errors in brackets. Standard errors are clustered on center level (*m* = 70)

* *p* ≤ 0.1, ** *p* ≤ 0.05, *** *p* ≤ 0.01

**Fig. 3:**
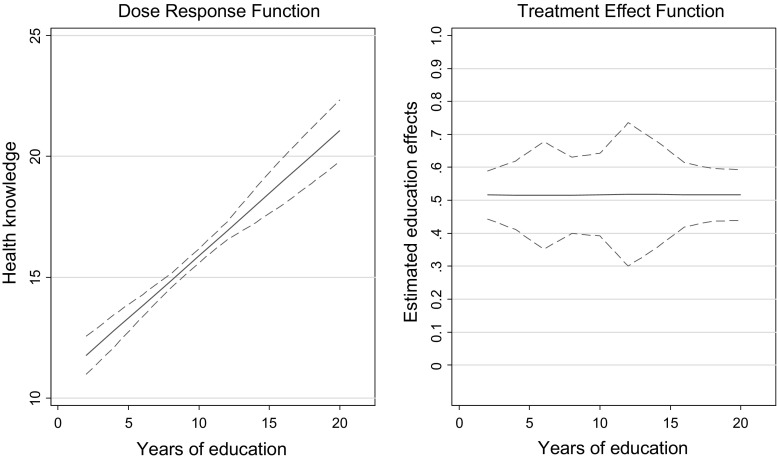
Dose response and treatment effect function: Education effects on health knowledge

Figure 3 illustrates the finding by plotting the dose response function and the treatment effect function over the treatment variable education. The estimated treatment effect function is flat, suggesting equal marginal gains in health knowledge for different education levels.

### Effects of education and health knowledge on health lifestyle

In the next step, we estimate the effects of education under control of the GPS first for the binary lifestyle variable (“Health lifestyle”) and then separately for the single health behavior indicators (“Estimation for separate health behaviors”). By additionally controlling for health knowledge in the models, we are able to assess the importance of this variable as a mediating factor.

#### Health lifestyle

Table 3 summarizes the results of the GPS-adjusted Logit and KHB estimations for the two binary lifestyle indicators (for full models including all pre-treatment controls see supplement S6.3). For both outcome variables we estimate first a baseline logit model (1a and 2a) with education as main explanatory variable, which is then extended by the knowledge index (1b and 2b). In a final step (1c and 2c), we further include an additional set of mediating variables that may potentially confound the estimation of knowledge effects. To ease the interpretation, all logit coefficients are presented as marginal effects calculated at the mean of the covariates.

We use the KHB method to decompose education effects in total, direct, and indirect effects operating through the health knowledge channel (full models in Table 9 in the appendix). While the total education effect is comparable to the one in the baseline logit estimation (1a and 2a), the indirect effect captures the share of the education effect on health lifestyle, which is driven by differences in knowledge levels. It is calculated by comparing the education coefficient in the harmonized baseline and the extended logit models. The larger the indirect relative to the total effect, the larger the explanatory power of the mediating variable. The strength of mediation is captured in Δ reported in the Table 3, which measures the percentage change of the education effect between the baseline and extended model. It can be interpreted as the share of the education effect, which can be explained with differences in knowledge levels.

**Table 3:** Logit models: education and knowledge effects on health lifestyle

	Outcome: health lifestyle					
	Cluster typologisation			Latent class typologisation		
	(1a)	(1b)	(1c)	(2a)	(2b)	(2c)
Years of education	0.035*** [0.006]	0.012* [0.006]	0.009 [0.007]	0.034*** [0.006]	0.010* [0.006]	0.010 [0.006]
Health knowledge		0.042*** [0.003]	0.041*** [0.003]		0.043*** [0.003]	0.042*** [0.004]
GPS	− 0.600 [0.382]	− 0.597 [0.389]	− 0.483 [0.369]	− 0.586 [0.360]	− 0.595 [0.36]	− 0.513 [0.349]
Wealth			0.062*** [0.017]			0.059*** [0.019]
Subjective health			0.012 [0.007]			0.014* [0.007]
Distance to health facility			0.003 [0.023]			0.005 [0.021]
Social support			0.172*** [0.038]			0.204*** [0.038]
Married			− 0.008 [0.032]			− 0.013 [0.033]
Children			− 0.007 [0.008]			0.002 [0.008]
Religiousness			− 0.029 [0.023]			− 0.019 [0.023]
Risk preferences			0.013** [0.006]			0.015*** [0.006]
Average education center			− 0.013 [0.020]			− 0.019 [0.020]
Average knowledge center			− 0.011 [0.015]			− 0.005 [0.015]
Average education peers			0.013 [0.015]			0.006 [0.016]
Average knowledge peers			− 0.009 [0.010]			− 0.005 [0.009]
KHB						
Δ (%)		64.7	68.6^a^		68.9	68.1^a^
Observations	1032	1032	1025	1041	1041	1034
Pseudo *R*^2^	0.035	0.134	0.171	0.034	0.139	0.179
AIC	1363.9	1227.7	1192.1	1382.4	1235.8	1195.5

Coefficients are displayed as marginal effects calculated at the mean of all covariates, standard errors in brackets. Standard errors are clustered at the center level (*m* = 70)

* *p* ≤ 0.1, ** *p* ≤ 0.05, *** *p* ≤ 0.01

^a^ Please note that to calculate the change in coefficients Δ for the models 1c and 2c, the extended model with knowledge and the other mediating factors is compared to a baseline model including all alternative mediating factors (not displayed in the table). All models control for area fixed effects

In the baseline specification, we find significant positive education effects for both lifestyle indicators and estimation procedures. Based on the logit estimation, an additional year of schooling raises the probability of having a healthy lifestyle by about 3.5% for both outcomes. The results suggest a statistically significant and meaningful effect of education on lifestyle under control for the GPS. The extended models (1b and 2b) reveal the important role of health knowledge as a mediating channel in the education–health lifestyle relationship. The knowledge index exhibits a significant positive effect on lifestyle. A one-point increase on the index is associated with a 4.2 and 4.3% increase in the chance of having a healthy lifestyle for the two outcome measures, respectively. Once health knowledge is included, education effects are considerably reduced in the models. Based on the KHB estimates, knowledge explains between 64.7 and 68.9% of the education effect, lending strong support to the allocative efficiency argument.

The strength of mediation does not considerably change once we control for other possible mediators, such as economic resources and risk preferences. Among these variables, we find wealth to have a significant and strong effect on health lifestyle. In model 1c an increase of one point on the wealth index increases the probability of having a healthy lifestyle by 6.2% (or 0.116 standard deviations with a one standard deviation increase in wealth). Using the KHB method, differences in wealth levels are found to explain about 32% of education effects, which is comparable to the mediation strength for wealth reported for instance in [14]. Although this is sizeable, knowledge can explain twice as much of the total education effect further underpinning the important role of this variable.

Apart from the influence of economic resources, we observe a positive effect of having social support in the community and of having a good subjective health status (only weakly significant in model 2c). Also, the risk preference measure has a positive effect. Surprisingly, respondents who are more risk-seeking, based on our 11-point scale self-assessment, are more likely to have a healthy lifestyle. This result may be due to the perception of risk in our sample, which consisted mostly of small business owners. Many of them seemed to perceive risk taking as a very positive personality trait and saw it as a sign of an entrepreneurial mindset. We do not find any evidence in our data for effects of peers’ average education and knowledge level on health lifestyle.

Table 4 shows the linear regression models with the continuous lifestyle indices as alternative outcomes (additional mediators not displayed). These additional results strongly confirm our previous findings. In the baseline models (1a and 2a) an additional year of schooling raises the lifestyle indices on average by 1.5 or 1.7% depending on the aggregation technique used. Once knowledge is introduced, the education effects are significantly reduced by between 53.3 and 60.1%. Like in the logit estimation, health knowledge exhibits a positive effect on the lifestyle outcomes. Note that while both the education and knowledge effects are smaller than the marginal effects calculated in the logit models, the estimated strength of mediation Δ is highly similar, reaffirming our previous conclusions.

**Table 4:** OLS models: education and knowledge effects on continuous outcomes

	Outcome: continuous health lifestyle					
	Additive index			Weighted index		
	(1a)	(1b)	(1c)	(2a)	(2b)	(2c)
Years of education	0.015*** [0.002]	0.007*** [0.002]	0.006*** [0.002]	0.017*** [0.002]	0.007*** [0.002]	0.006*** [0.002]
Health knowledge		0.016*** [0.001]	0.015*** [0.001]		0.019*** [0.001]	0.018*** [0.001]
GPS	− 0.268** [0.108]	− 0.267** [0.112]	− 0.261** [0.108]	− 0.313** [0.135]	− 0.312** [0.141]	− 0.297** [0.135]
Additional mediating factors	No	No	Yes	No	No	Yes
Change in coefficients^a^						
Δ (%)		53.3	56.7		58.8	60.1
Observations	1032	1032	1025	1032	1032	1025
Adjusted *R*^2^	0.085	0.26	0.303	0.073	0.251	0.298
AIC	− 1006.6	− 1225.2	− 1267	− 639.2	− 858.6	− 909.3

OLS coefficients with standard errors in brackets. Standard errors are clustered at the center level (*m* = 70)

* *p* ≤ 0.1, ** *p* ≤ 0.05, *** *p* ≤ 0.01

^a^ To calculate the change in coefficients for the linear OLS models, the baseline coefficient was subtracted from the one calculated in the extended model. The resulting term was then divided by the baseline coefficient to derive the percentage change in the baseline coefficient resulting from the inclusion of the mediator. Please note that to calculate the change in coefficients Δ for the models 1c and 2c, the extended model with knowledge and the other mediating factors is compared to the baseline model including all mediating factors (not displayed in the table). All models control for area fixed effects

#### Estimation for separate health behaviors

We repeat the analysis for the separate health behavior indicators, which were used as the basis for the lifestyle typologisation. Table 5 presents the results for the logit estimation with the single outcome indicators in the rows. Again, the baseline models (a) are extended by the knowledge index first without (b) and then under control for additional mediating factors (c).

**Table 5:** Logit models: baseline and extended models for separate health behaviors

Health behaviors	Years of education	Health knowledge	KHB Δ (%)	Other mediators included?	*N*	Pseudo *R*^2^	AIC
Exercising							
(1a)	0.009* [0.005]			No	1041	0.003	1189.2
(1b)	0.004 [0.006]	0.010** [0.004]	56.8	No	1041	0.01	1183.0
(1c)	0.002 [0.006]	0.008** [0.004]	73.0	Yes	1034	0.029	1178.4
Fruit consumption							
(2a)	0.023*** [0.006]			No	1039	0.014	1428.9
(2b)	0.016*** [0.006]	0.014*** [0.004]	32.8	No	1039	0.024	1416.6
(2c)	0.009 [0.006]	0.011*** [0.004]	38.6	Yes	1032	0.054	1389.4
Keeping a diet							
(3a)	0.010* [0.006]			No	1041	0.003	1343.0
(3b)	0.003 [0.006]	0.013*** [0.003]	69.3	No	1041	0.014	1331.1
(3c)	0.002 [0.006]	0.012*** [0.003]	78.4	Yes	1034	0.023	1334.6
Routine check last year							
(4a)	0.003 [0.005]			No	1041	0.005	1433.6
(4b)	− 0.009* [0.005]	0.023*** [0.004]	–	No	1041	0.031	1398.5
(4c)	− 0.012** [0.005]	0.021*** [0.004]	–	Yes	1034	0.058	1375.8
Routine check next year							
(5a)	0.019*** [0.005]			No	1041	0.016	1338.2
(5b)	0.013** [0.005]	0.012*** [0.003]	33.5	No	1041	0.024	1329.0
(5c)	0.014** [0.006]	0.012*** [0.004]	29.2	Yes	1034	0.046	1317.4
Healthcare provider							
(6a)	0.017*** [0.004]			No	1041	0.014	1374.4
(6b)	0.006 [0.005]	0.020*** [0.003]	62.8	No	1041	0.037	1345.1
(6c)	0.006 [0.006]	0.020*** [0.004]	64.6	Yes	1034	0.061	1327.2
Breast self-examination							
(7a)	0.032*** [0.006]			No	1041	0.046	1262.5
(7b)	0.014** [0.006]	0.034*** [0.003]	56.5	No	1041	0.121	1165.4
(7c)	0.014*** [0.005]	0.032*** [0.003]	53.6	Yes	1034	0.127	1174.4
Ever used family planning							
(8a)	0.003 [0.004]			No	1038	0.016	920.7
(8b)	− 0.002 [0.004]	0.009*** [0.003]	–	No	1038	0.028	911.3
(8c)	0.001 [0.004]	0.007** [0.003]	–	Yes	1031	0.089	877.1
Hand-washing							
(9a)	0.022*** [0.005]			No	1041	0.013	1424.0
(9b)	0.011** [0.005]	0.020*** [0.003]	48.9	No	1041	0.032	1398.4
(9c)	0.011* [0.006]	0.017*** [0.004]	42.4	Yes	1034	0.044	1396.5
Not drinking untreated water							
(10a)	0.030*** [0.006]			No	1041	0.039	1394.9
(10b)	0.030*** [0.006]	0.001 [0.004]	2.4	No	1041	0.039	1396.7
(10c)	0.022*** [0.006]	0.001 [0.005]	2.6	Yes	1034	0.049	1396.2
Public health insurance							
(11a)	0.012*** [0.005]			No	1038	0.017	1059.1
(11b)	0.003 [0.005]	0.018*** [0.004]	76.6	No	1038	0.048	1027.7
(11c)	0.003 [0.006]	0.019*** [0.004]	77.9	Yes	1031	0.056	1037.7
Search for information							
(12a)	0.022*** [0.005]			No	1041	0.02	1422.9
(12b)	0.009 [0.005]	0.024*** [0.004]	60.0	No	1041	0.049	1382.9
(12c)	0.008 [0.006]	0.024*** [0.004]	60.1	Yes	1034	0.086	1343.9
Learning new info about diseases							
(13a)	0.016*** [0.006]			No	1040	0.007	1410.0
(13b)	0.002 [0.006]	0.027*** [0.004]	87.1	No	1040	0.044	1359.6
(13c)	0.004 [0.006]	0.029*** [0.004]	76.8	Yes	1033	0.056	1358.7
Search info about family planning							
(14a)	0.001 [0.004]			No	1040	0.019	971.0
(14b)	− 0.001 [0.005]	0.005 [0.004]	–	No	1040	0.023	969.8
(14c)	0.004 [0.005]	0.005 [0.004]	–	Yes	1033	0.08	926.5

Logit coefficients in cells, standard errors in brackets. Standard errors are clustered on center level (*m* = 70). All models control for the GPS and area fixed effects. Additional mediators included in models c: wealth, subjective health, distance to health facility, social support, marital status, children in household, religiousness, risk preferences, average education and knowledge level in center and direct peer group. Please note that to calculate the change in coefficients Δ for the models c, the extended model with knowledge and the other mediating factors is compared to a baseline model including all alternative mediating factors

* *p* ≤ 0.1, ** *p* ≤ 0.05, *** *p* ≤ 0.01

For most of the considered indicators the findings are similar to the ones for the aggregated lifestyle measures. Education has a significantly positive effect on 11 of the 14 health behaviors. According to the baseline logit estimates, an additional year of schooling raises the probability for regular exercising by 0.9%, of regular fruit consumption by 2.3%, of having no problems with keeping a diet by 1.0%, of planning to undergo a routine check-up by 1.9%, of having a personal health care provider by 1.7%, of using breast self-examination by 3.2%, of using appropriate hand-washing practices by 2.2%, of not drinking untreated water by 3.0%, of having a personal health insurance by 1.2%, of having searched for health information by 2.2%, and of having learned new information about diseases by 1.6%.

Once knowledge as a potential mediator is controlled for, education effects are reduced in all models and even become insignificant in some cases. The health knowledge measure, on the other hand, exhibits a positive effect in most models, revealing a pattern that is consistent with the one observed for the aggregate lifestyle outcomes for 10 of the 14 indicators. Yet, some heterogeneity remains. In two cases, health knowledge exhibits a significant effect even though we do not find an education effect, suggesting that knowledge may also be of importance independent of education levels. Only in one case, the drinking of untreated water, variations in knowledge cannot explain the reported education effects.

Although the allocative efficiency explanation seems to matter for most of the considered indicators, the size of the education effects and the importance of knowledge as a mediator depends on the particular behavior. As expected from the changes in coefficients in the logit estimation, the KHB estimation confirms health knowledge to be an important mechanism in explaining education effects. According to the estimates from the full models including all mediators, between 29.2% (routine check-up next year) to 78.4% (Keeping a diet) of identified education effects are attributable to differences in knowledge.

As an additional robustness check, we also perform the analysis for selected health behaviors using specific knowledge measures that are closely related to and meaningful for the respective behavior (see supplement S6: Sensitivity tests). For instance, we test if respondents with knowledge about the benefits of exercising or healthy food consumption, e.g., in the prevention of hypertension, are more likely to show the beneficial behavior. Although the reduction in education effects is smaller in most cases when we use the specific knowledge measures (e.g., for exercising or public health insurance), we find a similar pattern as for the general knowledge index: Also under control for specific health knowledge, education effects are significantly reduced lending further support to our previous findings.

## Discussion

Our results confirm the main predictions of the allocative efficiency argument, which claims that (i) education raises a person’s health knowledge and that (ii) resulting differences in knowledge levels may explain differences in health lifestyle and behaviors across educational groups. The findings stand in stark contrast to the previous literature, which either suggests that education may not have a strong effect on knowledge [3, 37], or that knowledge only plays a minor role in the education–health lifestyle relationship [14, 41, 48].

Design and sampling differences might explain the diverging results: First, the focus of our study is poor households in the Philippines, a lower-middle-income economy. In this context, access to knowledge and information might be more restricted than in developed countries, where information is widely available and where public awareness is high [1, 17]. In our sample education may hence play a more decisive role for the dissemination of information and for raising awareness about health threats. Second, differences in the results of the studies may stem from the use of different study designs. For instance, in contrast to other studies, our knowledge measure relies on an extensive set of openly answerable questions about diverse health topics. With this procedure we attempted to capture the multidimensionality of the construct and to overcome common caveats found in the literature, such as measurement and interpretation issues.

Our study faces some limitations. First, due to the cross-sectional and non-experimental nature of our data, we are unable to make causal claims in our analysis. Although we try to control for pre-education characteristics using propensity scores, the results could be driven by simultaneity issues (e.g., those falling sick search for information and hence obtain more health knowledge), or omitted variables (unobserved characteristics, not captured in the propensity scores, such as preferences). Also, we can only provide an indirect test of the allocative efficiency mechanism, as we are not able to observe the full individual health production function including different input and outputs levels. People with more knowledge may adopt healthier lifestyles, but whether this is actually efficient, i.e. welfare/utility enhancing, is not evident from the analysis.

Another limitation is that our findings rely on survey data that might be prone to measurement and reporting errors. Also, in few cases we had to use proxy variables that can be particularly noisy. To check the reliability of our results we performed various consistency tests, for instance by using different model specifications and operationalizations, which did not indicate any problems with the used research design and identification strategy (see supplement S6: Sensitivity checks). Finally, the generalizability of our sample is restricted to members of our partner organization in the Philippines. Nevertheless, we believe that the sample is well-suited to test our theoretical predictions and represents an interesting case for the analysis of the allocative efficiency mechanism in a low-income setting. Further research using more representative data from different countries and regions is needed to test if the reported relationships hold in other settings and to further improve our understanding of determinants of health decisions in developing countries.

While we have focused on the link between education and knowledge in explaining differences in lifestyle across educational groups, there are other mediating channels, such as differences in economic resources and social capital, that can explain part of the education effects [14]. In our analysis, we controlled for these additional channels to determine the isolated mediation effect of health knowledge net of any other possible factors. Yet, due to data restrictions, we were not able to control for the full set of theoretically relevant mediators: For instance, in the economic literature time preferences are often named as important channel in explaining educational gradients in health [25, 55], although recent studies find that differences in tastes play only a minor role for the relationship between education and health outcomes [14].

Also, we are not able to properly control for changes in cognitive abilities that occurred as a result of schooling, although we include a measure of memorability in our propensity score estimation. Education may not only positively affect health knowledge and information, but also cognitive skills, enabling the better educated to process information more effectively [6, 9]. Put differently, it is less about what one knows, but more about how the knowledge is understood and translated into actions. At the same time, the results could be driven by cognitive dissonance [22, 24]: Those who express a certain negative health behavior may (unconsciously) avoid or suppress information that this behavior is bad (and vice versa for positive behavior). If this is the case, it is the respective health behavior that determines the information set, and not the other way around. In this context, beliefs may also play a role: In some cases, people may know about how a healthy lifestyle looks like, but they may not believe that strongly in the validity of the information.

We believe that a more in-depth exploration of these other potentially relevant pathways and mechanisms in the context of a developing country is promising. Also, we may miss an important part of the picture if we consider education effects only in terms of the quantity of education obtained (often measured in years of education), while ignoring the specific features of education and characteristics of the local context. Extending the perspective to such aspects can help us to understand not only how education shapes our behavior, but also what forms of education can be most effective in different situations.

## Conclusions

This paper provides an empirical test of the allocative efficiency hypothesis, which offers a behavioral explanation for the well-documented educational gradient in health [20]. The theory postulates that education raises health knowledge levels and, through this channel, positively impacts health lifestyle and ultimately health. Using original survey data from poor communities in the Philippines, we construct a comprehensive health knowledge index and consider respondents’ health lifestyles as an aggregate of 14 different context-relevant health practices, taking into account that single decisions are not made in isolation, but may depend on each other. Techniques of cluster and latent class analysis are used for the lifestyle typologisation.

Conditioning on pre-education characteristics, we find strong support for the predictions of the allocative efficiency hypothesis. Education exerts a positive effect on health knowledge levels, which are in turn positively related with health lifestyle. According to our estimates, an additional year of schooling increases the probability of having a healthy lifestyle by about 3.5%. Once knowledge as a potential mediating factor is introduced in the models, education effects are significantly reduced. Knowledge is found to explain between 65 and 69% of identified education effects on health lifestyle, more than any of the other considered mediating factors. The observed pattern does also hold, if we analyze the role of this potential mechanism separately for individual health behaviors. Even for the few behaviors, for which we do not detect any education effects, knowledge seems to play an important role, indicating that what one knows about health may also matter independent of education levels.

Our results suggest that schooling can generate important health externalities in poor communities. By raising general education levels, policy makers can contribute to improving knowledge and ultimately health outcomes through the allocative efficiency mechanism. Health deficits can be further reduced by implementing campaigns that directly address information gaps among the less educated [12, 13, 16]. On the other hand, simply providing information to the poor may not always be effective, as negative experiences with some information and education interventions have shown [5, 18, 31, 44]. The potential of an intervention largely depends on the specific context, the complexity of the provided health information, and the characteristics of the recipient [17]. Furthermore, although insufficient knowledge and information matter in many contexts, they are unlikely to be the only constraints to a healthy lifestyle, as we have highlighted in our analysis. Nevertheless, investments in education and knowledge can be a promising means to improve health in the long run and to potentially break health-related poverty traps that impede development in many regions.

### Electronic supplementary material

Below is the link to the electronic supplementary material.

Supplementary material 1 (DOCX 100 kb)

## Appendix

See Tables 6, 7, 8 and 9.

**Table 6:** Measurement of the health knowledge index and summary statistics

	Item/question	Accepted answers	Answer rule	Correct (%)
1	Do you know what an electro cardio graph (ECG) is and can you explain it to me?	Attach devices to the breast	R can describe method	61.2
2	What diseases can be detected with an ECG?	Heart diseases	R knows any disease	67.0
3	Do you know what a fasting blood sugar test is and can you explain it to me?	Blood sample is collected, need to fast before	R can describe method	59.5
4	What diseases can be detected with a blood sugar test?	Diabetes	R knows any disease	64.4
5	Do you know what a urinalysis is and can you explain it to me?	Collection and analysis of urine sample	R can describe method	80.6
6	What diseases can be detected with a urinalysis?	Diseases of inner organs, UTI, etc.	R knows any disease	81.1
7	Which family planning methods have you heard of?	Pill, IUD, injection implant, condom, sterilization, BBT rhythm, LAM, BOM, etc.	R knows > 3 methods	40.9
8	What can you do to improve the health of a newborn child?	Breastfeeding, immunization, right nutrition, check-up, proper hygiene, etc.	R knows > 2 methods	41.2
9	Do you know any techniques how mothers of newborn children can increase their milk production?	Nutrition, pump, massage, offering both breasts, skin-to-skin contact, keep schedule, etc.	R knows > 1 techniques	23.4
10	Do you know what breast self-examination is and can you explain it to me?^a^	Examination to detect changes or problems in the woman’s breast	R can describe method	60.1
11	Do you know a lot about healthy eating?	Agreement scale (1–5)	R agrees (4 or 5)	83.1
12	Do you know what urinary tract infection (UTI) is and can you explain it to me?	Painful inflammation of urinary tract	R can describe disease	83.0
13	Is UTI a contagious disease?	No		83.7
14	How can you prevent UTI?	Water consumption, not holding urine, hygiene, clothing, medicine, etc.	R knows > 1 methods	21.7
15	Do you know what Tuberculosis (TB) is and can you describe it to me?	Disease that usually attacks the lungs	R can describe disease	88.0
16	IS TB a contagious disease?	Yes		92.3
17	How much does a package of TB medicine cost at the health center?	Zero PHP (financed by national TB program)		45.8
18	Do you know what TB DOTS is and can you explain it to me?	Free and accessible medication, monitoring	R can describe program	33.5
19	What are symptoms of TB?	Coughing, chest/back pain, bloody sputum, fever/cold, weight loss, loss of appetite, fatigue, breathing/sleeping problems, skin/eye color, etc.	R knows > 3 symptoms	25.3
20	Do you know what hypertension is and can you explain it to me?	Constant high blood pressure		67.9
21	Is hypertension a contagious disease?	No		96.0
22	How can you prevent hypertension?	Diet, exercise, rest, avoid smoking/drinking, avoid heat, water, check-up, medicine, etc.	R knows > 2 method	22.7
23	Can you use antibiotics to reduce the harmful effects of a viral infection, such as a cold or flu?	No		26.7
24	What problems can occur if you take antibiotics too often?	Resistance, liver/kidney or other organ damage	R knows 1 side-effect	49.11
25	Do you know any diseases you can get from having sex?	AIDS/HIV, genital warts, gonorrhea, herpes,* Chlamydia*, syphilis, hepatitis, infection, vermin, etc.	R knows > 1 STD	43.8
26	Through which channels can you apply for a PhilHealth^b^ membership aside from going directly to the PhilHealth Office?	Health center, municipal office, barangay hall, work, online, etc.	R knows alternative channel	77.1
27	If you are PhilHealth member, who can be covered with you through your membership as beneficiary?	Children, spouse, parents	R knows > 1 beneficiary	63.9
28	Do you need to provide a birth certificate to become a PhilHealth member?	No		20.5

*R* respondent

^a^ We use breast self-examination as indicator, although studies have put into question the effectiveness of this method for early cancer screening and detection [62]. Despite this, the method is still being used in the Philippines and also promoted by our partner organization

^b^PhilHealth is the public health insurance in the Philippines

**Table 7:** Measurement of health behaviors and summary statistics

Health behavior	Measurement (all indicators binary coded: 1 if respondent shows behavior)	Percent of sample
Exercising	Respondent was physically active at least once a week	25.35
Fruit consumption	Respondent consumed fruits at least every second day a week	49.26
Keeping a diet	Measured based on respondents’ self-assessment. Respondents agreed that they do not have problems with keeping a diet	65.85
Routine check-up last year	Respondents underwent a routine check-up in the past 12 months without feeling sick or experiencing any disease symptoms	32.99
Routine check-up next year	Respondent planned to undergo a routine check-up as part of the health program provided by our partner organization in the next 12 months	64.94
Healthcare provider	Respondent had a personal health care provider, i.e. a health professional who knows her and her medical history well	61.94
Breast self-examination	Respondent performed breast self-examination in the past 3 months as an early breast cancer detection method for mature women	32.46
Ever used family planning	Respondent ever used reliable family planning methods (Pill, IUD, injection, implant, condom, sterilization, rhythm, body temperature, or BOM) in her life	83.77
Hand-washing	Respondents were asked at which occasions they wash their hands regularly. Answers were categorized based on recommendations given by the US Center for Disease Control and Prevention (e.g., before preparing food and eating, or after using the bathroom) Indicator takes the value one if respondent washed her hands on at least two of 7 recommended occasions	54.74
Not drinking untreated water	Respondent did not drink any untreated, unfiltered tab or dwell water in the past 3 days	47.41
Public health insurance	Respondent was personally insured with public health insurance (not considering beneficiaries of other family members)	20.94
Search for health information	Respondent actively sought information about health-related topics in the past 3 months	52.3
Learning info about diseases	Respondent obtained new information about health threats that she was not aware of in the past 6 months	41.4
Search info about family planning	Respondent actively sought information about family planning in the past 12 months either for herself or other family members	17.98

**Table 8:** OLS models: estimation of generalized propensity score

	Education
Age: < 35	− 0.650 [2.760]
Age: 35–44	0.080 [0.301]
Age: 45–54	0.041 [0.220]
Mother with secondary education	0.661*** [0.212]
Father with secondary education	0.811*** [0.207]
Mother not known	− 0.327 [0.563]
Father not known	− 0.681 [0.586]
Both parents literate	1.009*** [0.267]
Cognitive abilities	0.278*** [0.060]
Early work experience (≤ age of 10)	− 1.020*** [0.318]
Above median literacy rate in birth province (bp)	− 0.292 [0.303]
Above median population density rate in bp	0.051 [0.216]
Above median elementary school completion rate bp	0.628** [0.298]
Above median electrification rate in bp	− 0.244 [0.308]
Distance of bp to capital in 100 km	− 0.046 [0.042]
Constant	6.903*** [0.384]
Observations	1041
Adj. *R*^2^	0.135
AIC	5007.1

**Table 9:** KHB models: decomposing education effects on health lifestyle

	Cluster typologisation		Latent class typologisation	
	Without other mediators	With other mediators	Without other mediators	With other mediators
	(1b)	(1c)	(2b)	(2c)
Education effects				
Total effect	0.171*** [0.029]	0.157*** [0.032]	0.167*** [0.028]	0.164*** [0.031]
Direct effect	0.060* [0.031]	0.049 [0.034]	0.052* [0.030]	0.052 [0.034]
Indirect effect	0.111*** [0.014]	0.107*** [0.015]	0.115*** [0.015]	0.111*** [0.015]
Δ (%)	64.7	68.6	68.9	68.1
Observations	1032	1025	1041	1034

Cell entries are coefficients estimated with the KHB method. Standard errors in brackets are clustered at the center level. The total effect refers to the education effect in the baseline model; the direct effect refers to the effect in the extended model. The indirect effect, which is due to changes in the mediating factor (health knowledge) is the difference between the total and direct effects. Δ reports the percentage change in the education coefficient after controlling for the relevant mediator. Additional control variables are included in the models, but not displayed: Household size, number of children, marital and relationship status, neighborhood dummies. Models 1c and 2c additionally control for other mediating factors: Subjective health, distance to health facility, social support, religiousness, risk preferences, average education and knowledge in center, average education and knowledge in direct peer group

* *p* ≤ 0.1, ** *p* ≤ 0.05, *** *p* ≤ 0.01

## Footnotes

1 Several studies have tested for the role of health information and knowledge in developing countries [12, 13, 16]. They show that additional health-related information can lead to changes in behaviors, but its effectiveness strongly depends on the kind of information provided, the way of communicating it, and characteristics of the recipient, e.g., her absorptive capacity (see [17] for an overview).

2 For example, not knowing that smoking causes bladder cancer, as asked in the 1990 US National Health Interview Survey [14], does not mean that one does not know that smoking is harmful. At the same time, knowing that smoking causes cancer does not imply that one knows (or understands) that smoking is bad for health (see “Discussion” for a more lengthy discussion).

3 For instance, respondents were asked to explain the procedures of different medical tests. An answer was evaluated as correct, if the respondent was able to show that she possesses a basic understanding of the tests and could vaguely describe the procedure. For instance, as minimal answer to correctly describe what an electro cardio graph (ECG) is, respondents had to know that for this test devices are being attached to the breast.
