# Supplementary material for: The health knowledge mechanism: evidence on the link between education and health lifestyle in the Philippines
Source: Eur J Health Econ. 2018 Jan 3;20(1):27–43. doi: 10.1007/s10198-017-0950-2 (PMC6394601; doi:10.1007/s10198-017-0950-2)
Supplement: Supplementary file 1 — Supplementary material 1 (DOCX 100 kb) [file 10198_2017_950_MOESM1_ESM.docx]

Supplementary Material

The Health Knowledge Mechanism: Evidence on the Link between Education and Health Lifestyle in the Philippines

# Sample Selection and Data Collection

In total, 1064 individuals were interviewed for this study. The interviewed respondents lived in the greater area of Metro Manila and were part of microfinance centers which had up to 42 members, depending on the client base of our partner organization in the neighborhood. The sample selection was done using a multi-stage sampling procedure: In a first step, microfinance groups were randomly drawn as primary sampling units from the baseline population of centers in three wider geographical areas in Metro Manila and the neighboring province of Rizal. The three geographical areas were selected by our partner organization and represent the diverse social and geographical background of the organization. In a second step, a number of clients was randomly selected from each center (proportional to the total size of the group) for the interviews. Our research instrument consisted of different sections on participants’ health situation, her health behavior, her educational background, her financial situation, and other socio-demographic and household characteristics.

In addition, we collected information about respondents’ homes which was merged with geographical information about the location of public health facilities in the area. The infrastructure data was provided to us by the National Mapping and Resource Information Authority in the Philippines. (<http://www.namria.gov.ph/>). Furthermore, we derive information about the birth province of our respondents using data from the Philippine Census of Population and Housing for the years 1948, 1960, 1970, 1975, 1980, and 1990. The data was provided to us by the Philippine Statistical Authority and encoded by the authors. Provinces are the main political and administrative entities in the Philippines. In 2015 there were 81 provinces which formed the 18 greater regions (17 administrative and 1 autonomous regions) of the country.

The Philippines represent an interesting case for this study. Like other low and middle-income countries, they are characterized by strong inequalities in health care access and utilization with the poor being most strongly disadvantaged. Many of the most prevalent diseases could be effectively avoided or treated. Yet, the take-up of preventive and curative services as well as investments in health lifestyles are often low among the poor (WHO 2011; WHO 2013b). Besides supply-side restrictions in access to health care, lacking information and knowledge are named as primary reason for low health investments in the literature (Dupas 2011). Gaining a better understanding of the mechanisms that influence health-related decisions is hence not only of importance from an academic point of view, but also for the design of policies which aim at reducing informational and knowledge deficits among disadvantaged populations.

# Estimation Strategy

## Generalized Propensity Score Estimation

We are interested in estimating how different exposure to education influences a person’s level of knowledge and her health lifestyle as an aggregate measure of different health behaviors. We test for this relationship using propensity score matching (PSM) with years of education as a continuous treatment variable (Rosenbaum & Rubin 1983). Hirano & Imbens (2004) developed a generalization of the binary-treatment PSM method for continuous treatments with similar properties and assumptions (see also Egger & von Ehrlich 2013). The formalization is based on the potential outcome framework. For each unit $i$ in the sample there exists a potential outcome $Y_{i}\left( t \right)$ for treatment level $t \epsilon\mathcal{T}$ in the interval $[t_{0}, t_{1}]$. In the analysis we are interested in estimating the average *dose-response function* $\mu\left( t \right)= E[Y_{i}(t)]$ of education on health knowledge and health behavior based on a vector of covariates $X_{i}$, the actually observed treatment status $T_{i}\in[t_{0}, t_{1}]$, and the potential outcome resulting from the received treatment level $Y_{i}=Y_{i}(T_{i})$. In the implementation of the procedure the (potentially transformed) treatment variable is assumed to be normally distributed given the covariates.

The estimation of the dose response function requires the actual treatment level, conditional on the set of covariates X, to be independent of the potential outcome, such that:

| $Y_{i}\left( t \right)\perp T_{i} \vert X_{i} for all t \in T$ | (1.1) |
| --- | --- |

This so called weak unconfoundedness assumption is key for a causal interpretation of the results. Like in the binary treatment case it is not statistically testable. The assumption can be violated, if the potential outcome has an influence on the treatment level or if omitted variables influence both outcome and treatment. For instance, in our case, health in childhood or unobserved personality traits, such as time preferences (Fuchs 1982; van der Pol 2011), could influence both the propensity to get educated and health lifestyle. However, even if this assumption does not hold, the method can still be applied and useful in describing the relationship between the variables of interest.

With the conditional density of the treatment given the covariates $r\left( t, x \right)= f_{T|X}(t|x)$ the generalized propensity score (GPS) is defined as $R=r(T,X)$. The GPS can be described as an individual’s propensity to obtain a certain treatment level. It has a balancing property, which requires for educational strata with similar GPS that the probability of $T=t$ does not depend on the value of $X$. Combined with the unconfoundedness assumption, the balancing property implies that assignment to different treatment levels is unconfounded given the GPS. Furthermore, it is required that different treatment strata have common support. This means that there are individuals who, even if they are different in their treatment status, are comparable in terms of their GPS.

The GPS can be used to estimate the dose response function $\mu\left( t \right)$ by averaging the conditional expectation function $\beta(t, r)$ over the GPS at each treatment level.

| $\mu(t)= E[\beta(t,r(t,X))]$ | (1.2) |
| --- | --- |

The dose response function describes the effect of different doses of the treatment, in our case education, on the outcome variables. In the implementation of the approach in Section 4 we use a Stata user-written program by Bia & Mattel (2008), which also allows us to verify the main testable assumptions of the procedure.

## Dose Response Functions: Effects of Education on Health Knowledge and Lifestyle

We continue our empirical analysis with a test of the main predictions of the allocative efficiency argument. First, we estimate the dose response function for health knowledge as an outcome variable using linear OLS estimation with standard errors clustered at community level (m=70). We include the GPS in the estimation to condition on pre-education characteristics. Under the first prediction of the allocative efficiency hypothesis, we expect $\alpha_{1}$ to be significantly larger than zero.

| \| $K= \alpha_{0}+E\alpha_{1}+GPS\alpha_{2}$ \| \| --- \| | (2.1) |
| --- | --- | --- |

In the final step of our analysis, we are interested in the effect of education on health lifestyle and the role of knowledge as a mediating factor. Based on their behavior, respondents are categorized into two lifestyle types, a healthy and an unhealthy one. For the categorization we use 14 binary coded health behaviors with high relevance for the low-income context. The data aggregation facilitates the interpretation of our results and allows us to account for interdependencies across the different health behaviors. Methods of cluster analysis and latent class modeling are used to assign respondents to the two lifestyle types (Kaufman & Rousseeuw 2009; Hagenaars & McCutcheon 2009). While cluster analysis uses (arbitrary) distance and similarity criteria to determine the lifestyle typology, latent class analysis follows a probabilistic model-based approach. Applying both methods allows us to test for the robustness of our results using different typologisation techniques. For the cluster analysis we apply a k-mean (k=2) partition approach with the simple matching similarity coefficient used for the clustering. As the k-mean procedure does not allow for a deterministic classification, we rerun the method 2000 times and select the typologisation that maximizes the average difference across all behavioral indicators between the identified healthy and unhealthy lifestyle groups (see Table 1 in Section 3.3.3.). For the latent class analysis we use a user-written Stata command provided by the Penn State Methodology Center (Lanza et al. 2015).

Education effects are first estimated for the lifestyle typologisation categories and then separately for each health behavior to identify common patterns in the data. As both the health lifestyle and behavioral indicators (B) are binary coded, we use logit models for the identification of the dose response function, for which we include again the GPS. The baseline model is extended in two steps: First, we add only our health knowledge measure K as a potential mediator (2.3). If the predictions of the allocative efficiency hypothesis hold, we expect that (i) education positively affects health lifestyle ($\beta_{1}>0$), and that (ii) the inclusion of the potentially mediating knowledge measure significantly reduces the education effects ($\beta_{1}-\gamma_{1}>0)$.

| $P\left( B=1 \vert E, GPS, C \right)= \frac{exp(\beta_{0}+E\beta_{1}+GPS\beta_{2})}{1+exp(\beta_{0}+E\beta_{1}+GPS\beta_{2})}$ | (2.2) |
| --- | --- |
| $P\left( B=1 \vert E, GPS, C,K \right)= \frac{exp(\gamma_{0}+E\gamma_{1}+GPS\gamma_{2}+K\gamma_{3})}{1+exp(\gamma_{0}+E\gamma_{1}+GPS\gamma_{2}+K\gamma_{3})}$ | (2.3) |

In a final step (model not displayed), we extend the baseline model (2.2) by additionally including a set of other potential factors M which have been proposed as alternative mediating channels in the literature (Cutler & Lleras-Muney 2010; Fuchs 1982; Picone et al. 2004; Ettner 1996). Like before, we add knowledge K to the right-hand side of the baseline model to test for changes in the education coefficient. By including the other variables M, we can ensure that any change in the education effect is due to the inclusion of knowledge and not another potentially confounding mediating factor. In particular, we control for economic resources, risk preferences, access to health facilities, social capital, marital status, number of children in the household, religiosity, and the subjective health status. Furthermore, we control for the education and knowledge background in the microfinance network and in the direct peer group to take potential contextual peer effects into consideration (Durlauf & Ioannides 2010; Jackson 2011). These have been shown to be of importance for health behaviors in different other settings (Fletcher 2014). By controlling for these additional factors we are able to identify the mediation effect of health knowledge net of any other channels.

The comparison of education effects *(*$\beta_{1}-\gamma_{1}$*)* across non-linear models is not straightforward, as the scale of the underlying models may change if an additional factor is included. To be able to make cross-model comparisons, we apply the *KHB method* (Breen et al. 2013; Kohler et al. 2011), which harmonizes the scale of the baseline and full logit models in a two-step procedure. At first, the mediating variable K is regressed on the original explanatory variable E. The residual R of this first estimation capturing the unexplained variation in the mediator is then included in the baseline model (2.2). This leads to a scale harmonization between the baseline and the extended model including the mediator (2.3), making direct comparisons between the education coefficients possible. The difference in coefficients between both models can be considered as an indication for the strength of the mediation by the health knowledge variable. The KHB method takes the difference in coefficients and tests if it is significantly different from zero, which would support the allocative efficiency argument.

# Measurement of Pre-Education Characteristics

Education is measured in years of schooling in our study. All results are robust to the use of alternative education measures, such as the highest educational degree. The sample is relatively well educated. On average, respondents completed 9.6 years of education, which is comparable to the high national average of 8.9 years in the Philippines in 2013 (UNDP 2014). 7.2% of our sample has not completed primary education, 11.1% completed primary school, 48.6% visited or completed highschool, and 33.1% pursued some form of tertiary education, either vocational (10.12%) or at college (8.51% with completed degree, 14.47% without college degree).

We estimate respondent’s propensity to obtain a specific level of education using information on relevant personal pre-education characteristics: Parental education is measured with two dummies that capture if the father and mother visited or completed secondary school as well as a dummy that measures if both parents were able to read and write. Furthermore, we include two dummies measuring if a person grew up without a mother and/or father to capture effects of the missing parent. Furthermore, we include a dummy measuring if the respondent had early work experience at an age younger than 11 years as a proxy for her wealth in childhood. Finally, we control for respondent’s age which is measured in four categories (<35, 35-44, 45-54,>54).

Personal cognitive abilities were approximated by a brief word recall test (10 words). Memorability is closely related to what is referred to as fluid intelligence in the psychological literature, which is relatively stable over the life course under control for age (Conway et al. 2002; Oberauer et al. 2000; Kane et al. 2006; Deary et al. 2000). Still, we acknowledge that the inclusion of cognitive abilities in the propensity score estimation may be controversial as they can be both an input to and an outcome of education. Yet, as we show below our results are not sensitive to the use of different model specifications and operationalization procedures (see section S6). They do also not change if the indicator for cognitive abilities is removed from the estimation.

Besides personal characteristics, we include different potentially relevant birth province characteristics into the models.^[[1]](#footnote-1)^ We exploit the particularly rich variation in provinces of origin in our sample (respondents came from 68 different provinces, and only 25 % were born in one of the study areas in Metro Manila or Rizal Province). In particular, we are interested in context variables, which capture the economic development level and educational infrastructure in the province and are expected to influence individual education decisions. All data were derived from the Philippine Census of Population and Housing since 1948. Information on respondent’s birth province and birth year was merged with the environmental background characteristics of the respective province at the time of respondent’s birth. If the respondent’s birth year was in between two censuses, we used linear extrapolation to obtain an estimate for the considered variables in the birth year. Missing data for provinces in selected years was imputed based on regional averages. We use a broad variety of provincial background characteristics, namely, the distance of the birth province to the capital, the provincial literacy rate, the population density, the elementary school completion rate, and the electrification rate in the province. All variables, except distance to the capital, were dichotomized at the median^[[2]](#footnote-2)^.

# Measurement of Additional Mediating Factors

To identify the isolated mediation effect of health knowledge net of any other explanatory channels, we control for a broad set of other potentially mediating factors in our full model specification. We include such factors that may potentially be related to education, knowledge, and health lifestyle.

Economic resources were measured with an asset based wealth index which was generated by employing principal component analysis using the information on 28 different assets, such as vehicles or household appliances, (Vyas & Kumaranayake 2006). To assess subjective health status, which may influence current health lifestyle, respondents were asked how they rate their health on a scale from 0-10. Risk preferences were measured by asking respondents’ for their general willingness to take risks on a scale from 0-10 (Dohmen et al. 2011). In addition, we control for marital status and number of children in the household.

Access to the local health infrastructure was operationalized with the linear distance between the clients’ homes and the next health facility. To measure social support respondents were asked if there was at least one person among her friends and acquaintances who gave her advice on health, shared information, and encouraged her to undergo medical check-ups. The resulting indicator has a range from one to four. Subjective religiosity on a scale from 1-4 is used as an indicator for religiousness, an important cultural variable in the Philippines.

As a final critical mediating channel we control for the mean education and knowledge level in the whole microfinance group and in the respondent’s direct peer group. Both higher levels of education and knowledge in the network may make individuals more likely to express a healthy lifestyle in form of contextual or exogenous peer effects (Manski 1993; Fletcher 2014). At the same time, education and knowledge are likely to be correlated within the peer groups due to homophilous peer group formation, which may confound our estimation of individual knowledge effects, if not properly accounted for.

The direct peer group was identified based on a sociometric network questionnaire (Perkins et al. 2014) which consisted of four relationship questions in which respondents were asked to nominate those people in the microfinance group (i) who they perceived to be their personal friend, (ii) with who they had contact at least once a week, (iii) who they perceived as one of their 5 best friends, and (iv) who they shared private, intimate thoughts with. If a group member was nominated in either one of these questions, she was counted as a direct peer in our analysis. Although with this procedure we may not capture the entire social network of our respondents, the measure can serve as a proxy for the mean education and knowledge level among the peers in the area, especially since for many of our respondents there is a strong overlap between the general social network and the microfinance group network (e.g. on average respondents have 2 of their 5 closest friends in the microfinance group).

# GPS Estimation and Test of Assumptions

In the estimation of the generalized propensity scores we closely follow the procedures outlined in Hirano and Imbens (2004) and Kluve et al. (2012). After estimating the GPS, the central assumptions of the propensity score method are tested: the normality assumption, the balancing of covariates along the treatment spectrum, and the common support condition.

## GPS Estimation

For the calculation of the GPS the treatment variable, years of education, which is not significantly different from a normal distribution according to a one-sample Kolmogorov-Smirnov test, is regressed on various relevant individual and environmental pre-treatment characteristics. Table S1 summarizes the results of the linear GPS estimation. Although the model explains a significant share of the variation in schooling, it is not able to exhaustively capture the selection into education challenging a causal interpretation of the estimated effects.

Table S1 - OLS models: Estimation of generalized propensity score

|  | Education | |
| --- | --- | --- |
| Age: <35 | -0.650 | [2.760] |
| Age: 35 - 44 | 0.080 | [0.301] |
| Age: 45 - 54 | 0.041 | [0.220] |
| Mother with secondary education | 0.661*** | [0.212] |
| Father with secondary education | 0.811*** | [0.207] |
| Mother not known | -0.327 | [0.563] |
| Father not known | -0.681 | [0.586] |
| Both parents literate | 1.009*** | [0.267] |
| Cognitive abilities | 0.278*** | [0.060] |
| Early work experience (≤ age of 10) | -1.020*** | [0.318] |
| Above median literacy rate in birth province (bp) | -0.292 | [0.303] |
| Above median population density rate in bp | 0.051 | [0.216] |
| Above median elementary school completion rate bp | 0.628** | [0.298] |
| Above median electrification rate in bp | -0.244 | [0.308] |
| Distance of bp to capital in 100 km | -0.046 | [0.042] |
| Constant | 6.903*** | [0.384] |
| Observations | 1041 | |
| Adj. R² | 0.135 | |
| AIC | 5007.1 | |

Notes: Coefficients in cells, standard errors in brackets. Standard errors are clustered on center level (m=70). P-value: * p≤0.1, ** p≤0.05, *** p≤0.01.

Parental education level and literacy exert strong effects on children’s education. Having literate parents, who obtained at least some form of secondary education, raises a person’s education level on average by almost 2.5 years. Furthermore, we find that respondents, who made early work experiences (at an age younger than 11) and who hence grew up in an economically less fortunate environment, had a significantly smaller chance of obtaining higher education degrees. Besides parental background characteristics, a person’s memorability is associated with higher educational attainment. A one point increase on the scale is associated with a significant average increase in years of schooling of 0.28. Interestingly, we do not find an effect of age on education, which might be due to early school reforms in the Philippines, which led to improved schooling access for large parts of the population. Among the provincial background characteristics, we find that respondents from provinces with a below median elementary school completion rate had a significantly lower education level, which might be due to differences in schooling infrastructures across the provinces. We do not find statistically significant effects for any of the other provincial background variables.

## Test of Main Assumptions

For the test of the balancing property, we divide the range of the treatment variable into three intervals. Following Hirano & Imbens (2004), we define the groups to have approximately an equal size: The first group ranges from 0 to 9 years of education and covers 36.95% of the sample (approximately up to lower secondary), the second group includes people with 10 to 11 years of education and makes up for 38.55% of the sample (approximately upper secondary and secondary completed), and the third group consists of persons with more than 11 years of education covering 24.51% (approximately respondents with tertiary education). For each individual we calculate the GPS at the median of the three groups. The groups are split into deciles based on individuals’ estimated GPS. The balancing property of this adjusted sample is then tested by comparing respondents in the first treatment group, i.e. those with low education, with respondents from the other two treatment groups, who belong to the same GPS decile. Differences across groups are analyzed with t-tests, based on weighted mean differences between the groups. Table S2 presents the results of the comparison. If the sample is well-balanced, we expect no significant differences across the educational strata. Indeed, for all considered covariates we observe no t-statistic larger than 1.64, providing strong evidence in favor of the balancing property for our sample.

Table S2 - GPS Matching: Test of balancing property in adjusted sample

|  | Interval 1  [0-9] | | Interval 2  [9.5-11] | | Interval 3  [11.5-19] | |
| --- | --- | --- | --- | --- | --- | --- |
| Age: 20 -34 | -0.0015 | [-0.7750] | 0.0013 | [0.5695] | 0.0011 | [0.3439] |
| Age: 35 - 44 | -0.0004 | [-0.016] | -0.0207 | [-0.9468] | 0.0178 | [0.7296] |
| Age: 45 - 54 | -0.0074 | [-0.2379] | 0.0277 | [0.9435] | -0.0165 | [-0.4681] |
| Reference: Age ≥ 55 |  |  |  |  |  |  |
| Mother with secondary education | -0.0031 | [-0.1411] | 0.0097 | [0.3577] | -0.0196 | [-0.7133] |
| Father with secondary education | -0.0081 | -0.3575] | 0.0136 | [0.4979] | -0.0168 | [-0.6059] |
| Mother not known | 0.0053 | [0.5098] | -0.006 | [-0.5185] | 0.0021 | [0.1360] |
| Father not known | -0.0022 | [-0.2146] | 0.0068 | [0.6393] | 0.0026 | [0.1726] |
| Cognitive abilities | 0.1149 | [1.4485] | -0.1268 | [-1.462] | -0.0553 | [-0.5836 |
| Early work experience (≤ 10) | -0.0074 | [-0.4517] | 0.0123 | [0.7356] | -0.0089 | [-0.3970] |
| Both parents literate | -0.0108 | [-0.6756] | 0.0074 | [0.4093] | -0.0179 | [-0.6900] |
| Above median literacy rate in birth province | 0.0249 | [0.7857] | -0.0200 | [-0.6763] | -0.0216 | [-0.5849] |
| Above median density rate in birth province | 0.0308 | [0.9576] | -0.0250 | [-0.8111] | -0.0014 | [-0.0389] |
| Above median elementary school completion rate in birth province | 0.0147 | [0.4770] | -0.0071 | [-0.2300] | -0.0202 | [-0.5635] |
| Above median electrification rate in birth province | 0.0129 | [0.4024] | -0.0189 | [-0.6053] | 0.0046 | [0.1244] |
| Distance of birth province to capital in 100 km | -18.093 | [-1.078] | 1.1925 | [0.0740] | 24.079 | [1.2450] |

Notes: Cell entries are aggregated differences between the GPS quintiles in one treatment stratum and the quintiles in the other two strata; t-values in brackets. P-value: * p≤0.1, ** p≤0.05, *** p≤0.01.

Finally, we test for the common support condition by graphically comparing the distribution of the GPS evaluated at the median of each interval between the three educational strata. For this, we first evaluate the GPS for the entire sample at the group median of the first educational interval. Then we plot the GPS distribution of the first treatment stratum against the GPS distribution of the other two treatment strata. This exercise is repeated for the second and third group. The histograms in Figure S1 show the distribution of the GPS for each educational group in red bars, plotted against the aggregate GPS distribution of the other two strata in blue bars. By inspecting the overlap of the distributions, we are able to examine the common support condition (see Kluve et al. (2012) and Flores et al. (2012) who use the same procedure). The histograms show that the common support condition is fulfilled, with the exception of minor deviations in the lower tail of the GPS distribution in the second educational stratum.

Figure S1 - Graphical inspection of common support condition

# Sensitivity Tests

We run various robustness checks to test whether our results are sensitive to the use of alternative measurements, model specifications, and estimation procedures. In particular, we try to replicate our main results using differently aggregated health knowledge indices (S6.1), analyzing the influence of knowledge indicators that are specifically relevant for selected health behaviors (S6.2), and employing nearest neighbor matching and models which do not only control for the GPS as aggregate measure, but for the full set of different pre-treatment background variables (S6.3).

## Alternative Aggregation of Knowledge Index

In our main models, we use a health knowledge index for which the single items were additively combined (scale from 0 to 28, mean of 15.7). Table S3 shows the results for the main models using alternative aggregation procedures for this key explanatory variable: (i) an additive index of the normalized knowledge items k where each item $k_{j}\in\left\{ 0\left. ,1 \right\} \right.$ was transformed by subtracting the mean of correct answers of all respondents and dividing it by the standard deviation (*standardized knowledge index*), and (ii) an index for which single items were weighted based on principal component analysis (*weighted knowledge index*).

As can be inferred from the table, the results largely confirm the findings of our main analysis. The inclusion of the health knowledge measures leads to a significant reduction in education effects. In all models, health knowledge has a substantial effect on health lifestyle. For instance, a one point increase on the standardized index is associated with a 1.8% increase in the probability of having a healthy lifestyle in the full model specification (1/2c) for the cluster typologisation.

Table S3 - Logit models: Effect of education and knowledge on health lifestyle

|  | **Outcome: health lifestyle** | | | | | |
| --- | --- | --- | --- | --- | --- | --- |
|  | Cluster typologisation | | | Latent class typologisation | | |
|  | (1a) | (1b) | (1c) | (2a) | (2b) | (2c) ^a^ |
| **(i) Standardized knowledge index** |  |  |  |  |  |  |
| Years of education | 0.035*** | 0.012** | 0.01 | 0.034*** | 0.011* | 0.01 |
|  | [0.006] | [0.006] | [0.007] | [0.006] | [0.006] | [0.006] |
| Health knowledge |  | 0.018*** | 0.018*** |  | 0.019*** | 0.018*** |
|  |  | [0.001] | [0.001] |  | [0.001] | [0.002] |
| GPS | -0.600 | -0.606 | -0.495 | -0.586 | -0.605* | -0.526 |
|  | [0.382] | [0.388] | [0.368] | [0.360] | [0.364] | [0.349] |
|  |  |  |  |  |  |  |
| **KHB** |  |  |  |  |  |  |
| Δ |  | 64.4% | 68.3% ^a^ |  | 68.7% | 67.8% ^a^ |
| Observations | 1032 | 1032 | 1025 | 1041 | 1041 | 1034 |
| Pseudo R² | 0.035 | 0.135 | 0.172 | 0.034 | 0.14 | 0.179 |
| AIC | 1364 | 1226 | 1191.1 | 1382.5 | 1233.8 | 1194.3 |
|  |  |  |  |  |  |  |
|  | **Outcome: health lifestyle** | | | | | |
|  | Cluster typologisation | | | Latent class typologisation | | |
|  | (1a) | (1b) | (1c) | (2a) | (2b) | (2c) ^a^ |
| **(ii) Weighted knowledge index (PCA)** |  |  |  |  |  |  |
| Years of education | 0.035*** | 0.011* | 0.008 | 0.034*** | 0.009 | 0.009 |
|  | [0.006] | [0.006] | [0.006] | [0.006] | [0.006] | [0.006] |
| Health knowledge |  | 0.210*** | 0.206*** |  | 0.216*** | 0.209*** |
|  |  | [0.014] | [0.016] |  | [0.015] | [0.017] |
| GPS | -0.600 | -0.571 | -0.451 | -0.586 | -0.565 | -0.479 |
|  | [0.382] | [0.383] | [0.367] | [0.360] | [0.357] | [0.345] |
|  |  |  |  |  |  |  |
| **KHB** |  |  |  |  |  |  |
| Δ |  | 69.3% | 72.3% ^a^ |  | 73.7% | 71.7% ^a^ |
| Observations | 1032 | 1032 | 1025 | 1041 | 1041 | 1034 |
| Pseudo R² | 0.035 | 0.138 | 0.175 | 0.034 | 0.142 | 0.182 |
| AIC | 1364 | 1221.3 | 1187 | 1382.5 | 1230.2 | 1190.9 |

## Specific Health Knowledge Measures

While we focus in our main analysis on the effect of general health knowledge, we reran our analysis to test whether respondents with specific health knowledge, e.g. about strategies to prevent hypertension, are more likely to undertake specific beneficial behaviors, such as exercising or healthy food consumption. The specific knowledge measures were generated using information from different (partly combined) items. All specific knowledge measures are either binary or dichotomized at the median (the following numbers in parentheses refer to the used items, see table A1 in appendix):

1) Exercising: Respondent was aware that regular exercising helps preventing hypertension (22)

2) Fruit consumption: Respondents agreed that they know a lot about healthy eating (11)

3) Keeping a diet: Respondent knew that keeping the right diet and maintaining a good weight can help preventing hypertension (22)

4) & 5) Check-up: Knowledge about lab tests (1-6). The 6 items were summed up and dichotomized at the median

6) Breast self-examination: Respondents could explain what breast self-examination is (10)

7) Family planning: Respondent could name at least one family planning method (7)

8) Public health insurance: Knowledge about insurance (26-28). The 3 items were summed up and dichotomized at the median.

Table S4 show the results of logit models in which we regress different binary coded health behaviors on specific health knowledge items which are of relevance for the particular behavior. Although the reduction in education effects is smaller in most cases when we use the specific knowledge measures (e.g. for exercising or public health insurance), we find a similar pattern as for the general knowledge index: Also under control for specific health knowledge, education effects are significantly reduced lending further support to our previous findings.

Table S4 - Logit models: Consistency check for specific health knowledge indicators

| Health behaviors |  | Years of education | | Specific health knowledge | | KHB Δ | Other mediators included? | N | Pseudo R² | AIC |
| --- | --- | --- | --- | --- | --- | --- | --- | --- | --- | --- |
| Exercising | (1a) | 0.009* | [0.005] |  |  |  | No | 1041 | 0.003 | 1189.2 |
|  | (1b) | 0.008 | [0.005] | 0.091*** | [0.028] | 13.1% | No | 1041 | 0.011 | 1182.2 |
|  | (1c) | 0.005 | [0.005] | 0.086*** | [0.028] | 14.3% | Yes | 1034 | 0.031 | 1175.9 |
| Fruit consumption | (2a) | 0.023*** | [0.006] |  |  |  | No | 1039 | 0.014 | 1428.9 |
|  | (2b) | 0.023*** | [0.006] | 0.083** | [0.041] | 1.4% | No | 1039 | 0.017 | 1426.8 |
|  | (2c) | 0.014** | [0.006] | 0.062 | [0.041] | 2.3% | Yes | 1032 | 0.049 | 1395.5 |
| Keeping a diet | (3a) | 0.010* | [0.006] |  |  |  | No | 1041 | 0.003 | 1343 |
|  | (3b) | 0.009* | [0.005] | 0.123*** | [0.037] | 8.6% | No | 1041 | 0.01 | 1336 |
|  | (3c) | 0.007 | [0.006] | 0.114*** | [0.036] | 7.9% | Yes | 1034 | 0.02 | 1337.8 |
| Routine check  last year | (4a) | 0.003 | [0.005] |  |  |  | No | 1041 | 0.005 | 1433.6 |
|  | (4b) | -0.006 | [0.005] | 0.193*** | [0.026] | - | No | 1041 | 0.031 | 1397.4 |
|  | (4c) | -0.008 | [0.005] | 0.160*** | [0.028] | - | Yes | 1034 | 0.056 | 1379.2 |
| Routine check  next year | (5a) | 0.019*** | [0.005] |  |  |  | No | 1041 | 0.016 | 1338.2 |
|  | (5b) | 0.015*** | [0.005] | 0.093*** | [0.031] | 22.8% | No | 1041 | 0.023 | 1330.8 |
|  | (5c) | 0.016*** | [0.005] | 0.095*** | [0.033] | 18.3% | Yes | 1034 | 0.045 | 1317.6 |
| Breast self-  examination | (6a) | 0.032*** | [0.006] |  |  |  | No | 1041 | 0.046 | 1262.5 |
|  | (6b) | 0.010** | [0.005] | 0.430*** | [0.023] | 66.8% | No | 1041 | 0.254 | 990.9 |
|  | (6c) | 0.009* | [0.005] | 0.420*** | [0.023] | 69.0% | Yes | 1034 | 0.262 | 997.9 |
| Ever used family planning | (7a) | 0.003 | [0.004] |  |  |  | No | 1038 | 0.016 | 920.7 |
|  | (7b) | 0.001 | [0.004] | 0.110*** | [0.023] | - | No | 1038 | 0.038 | 902.2 |
|  | (7c) | 0.002 | [0.004] | 0.081*** | [0.025] | - | Yes | 1031 | 0.095 | 871.6 |
| Public health insurance | (8a) | 0.012*** | [0.005] |  |  |  | No | 1038 | 0.017 | 1059.1 |
|  | (8b) | 0.011** | [0.005] | 0.074*** | [0.028] | 13.5% | No | 1038 | 0.024 | 1053.1 |
|  | (8c) | 0.010* | [0.005] | 0.080*** | [0.028] | 14.2% | Yes | 1031 | 0.035 | 1059.4 |

Notes: Logit coefficients in cells, standard errors in brackets. All coefficients are displayed as marginal effects calculated at the mean of all covariates. Standard errors are clustered on center level (m=70). All models control for the GPS and area fixed effects. Additional mediators included in models c: wealth, subjective health, distance to health facility, social support, marital status, number of children, religiousness, risk preferences, average education and knowledge level in center and direct peer group. P-value: * p≤0.1, ** p≤0.05, *** p≤0.01.

## Alternative Estimation Procedures

As a final robustness check, we use alternative estimation procedures to test for the consistency of our findings. First, we re-estimate our lifestyle models controlling for the full set of pre-education characteristics instead of the GPS as an aggregate control variable. Second, we employ nearest neighbor matching, for which the outcome of respondents is directly compared to the outcome of another (or several other) individual(s) (or nearest neighbors), who share similar pre-education characteristics.

Table S5 shows the lifestyle models, controlling for the full set of pre-education characteristics. Again, we regress the two binary lifestyle typologies on our two main explanatory variables, education and health knowledge. The estimation yields results that are highly similar to those obtained in our main analysis.

Table S6 shows the results for the nearest neighbor matching. For the matching we created a low and high education category based on respondents’ highest educational degree. In our specification, respondents with up to lower secondary education (completed 1^st^ or 2^nd^ year of high school) form the lower (30.5%) and respondents with at least upper secondary education (completed 3^rd^ or 4^th^ year of high school) the high education category (69.5%). To carry out our mediation analysis we manually implement the matching. We run regressions on a matched sample by re-weighting each case depending on the number of matched observations (University of Wisconsin Madison 2015). While this allows us to perform our mediation analysis, the standard errors may be incorrect and should be treated with care, as the procedure does not take the matching stage into account. Still, the results can serve as an indication for the meaningfulness of our previous findings.

Based on our estimates, being part of the high education group raises the probability of having a healthy lifestyle by 17.6% (1a) or 15.7% (2a). As the average difference in years of schooling between the two groups is about 4.8 years, these coefficients correspond very well with our previous findings in which the increase in the probability was estimated at 3.5% with every additional year of schooling. Similar to our previous models, the education effects are largely reduced once the knowledge index is controlled for. Using the nearest neighbor matching we can confirm the importance of knowledge as a mediating variable in the education-health lifestyle relationship.

Table S5 - Logit models: Effect of education and knowledge on health lifestyle under control for pre-treatment background characteristics

|  | **Outcome: health lifestyle** | | | | | |
| --- | --- | --- | --- | --- | --- | --- |
|  | Cluster typologisation | | | Latent class typologisation | | |
|  | (1a) | (1b) | (1c) | (2a) | (2b) | (2c) ^a^ |
| Years of education | 0.036*** | 0.014** | 0.011 | 0.034*** | 0.012* | 0.012* |
|  | [0.007] | [0.007] | [0.007] | [0.006] | [0.006] | [0.007] |
| Health knowledge |  | 0.042*** | 0.042*** |  | 0.043*** | 0.042*** |
|  |  | [0.003] | [0.004] |  | [0.003] | [0.004] |
|  |  |  |  |  |  |  |
| **Pre-treatment controls** |  |  |  |  |  |  |
| Age: 20 -34 | -0.065 | 0.021 | 0.065 | -0.06 | 0.027 | 0.057 |
|  | [0.066] | [0.060] | [0.065] | [0.070] | [0.064] | [0.068] |
| Age: 35 - 44 | -0.02 | -0.024 | -0.003 | -0.004 | -0.009 | 0.001 |
|  | [0.049] | [0.045] | [0.052] | [0.051] | [0.048] | [0.050] |
| Age: 45 - 54 | 0.028 | 0.02 | 0.025 | 0.042 | 0.035 | 0.037 |
|  | [0.046] | [0.041] | [0.038] | [0.043] | [0.040] | [0.037] |
| Mother with secondary education | -0.097*** | -0.085*** | -0.083*** | -0.075** | -0.062** | -0.060* |
|  | [0.033] | [0.029] | [0.030] | [0.035] | [0.031] | [0.031] |
| Father with secondary education | 0.079** | 0.074** | 0.051 | 0.054 | 0.05 | 0.027 |
|  | [0.039] | [0.035] | [0.034] | [0.038] | [0.035] | [0.033] |
| Mother not known | 0.066 | 0.066 | 0.061 | 0.022 | 0.022 | 0.017 |
|  | [0.113] | [0.108] | [0.111] | [0.112] | [0.109] | [0.112] |
| Father not known | -0.214* | -0.169* | -0.164* | -0.15 | -0.103 | -0.092 |
|  | [0.112] | [0.095] | [0.089] | [0.108] | [0.091] | [0.083] |
| Cognitive abilities | 0.015 | -0.01 | -0.01 | 0.021* | -0.004 | -0.002 |
|  | [0.012] | [0.012] | [0.012] | [0.013] | [0.012] | [0.012] |
| Early work experience (≤ 10) | 0.063 | 0.046 | 0.028 | 0.055 | 0.037 | 0.023 |
|  | [0.062] | [0.057] | [0.055] | [0.059] | [0.053] | [0.051] |
| Both parents literate | 0.014 | 0.026 | 0.023 | -0.002 | 0.007 | 0.001 |
|  | [0.041] | [0.042] | [0.041] | [0.037] | [0.036] | [0.038] |
| Above median literacy rate in birth province | -0.037 | -0.045 | -0.037 | -0.055 | -0.056 | -0.046 |
|  | [0.050] | [0.046] | [0.045] | [0.049] | [0.046] | [0.045] |
| Above median density rate in birth province | 0.026 | 0.016 | 0.01 | 0.02 | 0.012 | 0.005 |
|  | [0.041] | [0.039] | [0.037] | [0.039] | [0.037] | [0.035] |
| Above median elementary school completion rate in birth province | -0.055 | -0.063 | -0.072* | -0.005 | -0.014 | -0.023 |
|  | [0.050] | [0.047] | [0.042] | [0.053] | [0.048] | [0.043] |
| Above median electrification rate in birth province | 0.022 | 0.042 | 0.049 | -0.007 | 0.006 | 0.01 |
|  | [0.064] | [0.057] | [0.058] | [0.055] | [0.050] | [0.051] |
| Distance of birth province to capital in 100 km | -0.003 | 0.001 | 0.001 | -0.009 | -0.005 | -0.005 |
|  | [0.008] | [0.008] | [0.008] | [0.008] | [0.007] | [0.007] |
| Additional mediating factors | No | No | Yes | No | No | Yes |
| **KHB** |  |  |  |  |  |  |
| Δ |  | 60.1% | 65.1% ^a^ |  | 65.1% | 64.5% ^a^ |
| Observations | 1032 | 1032 | 1025 | 1041 | 1041 | 1034 |
| Pseudo R² | 0.049 | 0.144 | 0.18 | 0.045 | 0.143 | 0.183 |
| AIC | 1372 | 1240.7 | 1207.5 | 1394.8 | 1257.3 | 1217.7 |

Notes: Coefficients are displayed as marginal effects calculated at the mean of all covariates, standard errors in brackets. Standard errors are clustered at the center level (m=70). ^a^ Please note that to calculate the change in coefficients Δ for the models 1c and 2c, the extended model with knowledge and the other mediating factors is compared to the baseline model including all mediating factors (not displayed in the table). All models control for area fixed effects. P-value: * p≤0.1, ** p≤0.05, *** p≤0.01.

Table S6 – Nearest neighbor matching with weighting

|  | **Outcome: health lifestyle** | | | | | |
| --- | --- | --- | --- | --- | --- | --- |
|  | Cluster typologisation | | | Latent class typologisation | | |
|  | (1a) | (1b) | (1c) | (2a) | (2b) | (2c) ^a^ |
| High education | 0.176*** | 0.060 | 0.069 | 0.157*** | 0.063 | 0.073* |
|  | [0.042] | [0.044] | [0.045] | [0.049] | [0.046] | [0.041] |
| Health knowledge |  | 0.042*** | 0.043*** |  | 0.043*** | 0.043*** |
|  |  | [0.004] | [0.005] |  | [0.004] | [0.004] |
| Additional mediating factors | No | No | Yes | No | No | Yes |
| Observations | 1436 | 1436 | 1427 | 1450 | 1450 | 1444 |
| Pseudo R² | 0.036 | 0.131 | 0.167 | 0.02 | 0.154 | 0.188 |
| AIC | 1845.2 | 1661 | 1608.6 | 1900.9 | 1647.6 | 1600.6 |

Notes: Coefficients are displayed as marginal effects calculated at the mean of all covariates, standard errors in brackets. Standard errors are clustered at the center level (m=70). ^a^ Please note that to calculate the change in coefficients Δ for the models 1c and 2c, the extended model with knowledge and the other mediating factors is compared to the baseline model including all mediating factors (not displayed in the table). All models control for area fixed effects. P-value: * p≤0.1, ** p≤0.05, *** p≤0.01.

# References

Bia, M. & Mattel, A., 2008. A Stata package for the estimation of the dose-response function through adjustment for the generalized propensity score. *Stata Journal*, 8, pp.354–373.

Breen, R., Karlson, K.B. & Holm, A., 2013. Total, Direct, and Indirect Effects in Logit and Probit Models. *Sociological Methods & Research*, 42(2), pp.164–191.

Conway, A.R.A., Cowan, N., Bunting, M.F., Therriault, D.J. & Minkoff, S.R.B., 2002. A latent variable analysis of working memory capacity, short-term memory capacity, processing speed, and general fluid intelligence. *Intelligence*, 30(2), pp.163–183.

Cutler, D.M. & Lleras-Muney, A., 2010. Understanding differences in health behaviors by education. *Journal of Health Economics*, 29(1), pp.1–28.

Deary, I.J., Whalley, L.J., Lemmon, H., Crawford, J.R. & Starr, J.M., 2000. The stability of individual differences in mental ability from childhood to old age: Follow-up of the 1932 Scottish mental survey. *Intelligence*, 28(1), pp.49–55.

Dohmen, T., Falk, A., Huffman, D., Sunde, U., Schupp, J. & Wagner, G.G., 2011. Individual risk attitudes: Measurement, determinants, and behavioral consequences. *Journal of the European Economic Association*, 9, pp.522–550.

Durlauf, S.N. & Ioannides, Y.M., 2010. Social Interactions. *Annual Review of Economics*, 2(1), pp.451–478.

Egger, P.H. & von Ehrlich, M., 2013. Generalized propensity scores for multiple continuous treatment variables. *Economics Letters*, 119, pp.32–34.

Ettner, S.L., 1996. New evidence on the relationship between income and health. *Journal of Health Economics*, 15, pp.67–85.

Fletcher, J.M., 2014. Peer Effects in Health Behaviors. In A. J. Culyer, ed. *Encyclopedia of Health Economics*. pp. 467–472.

Flores, C. a., Flores-Lagunes, A., Gonzalez, A. & Neumann, T.C., 2012. Estimating the Effects of Length of Exposure to Instruction in a Training Program: The Case of Job Corps. *Review of Economics and Statistics*, 94(1), pp.153–171.

Fuchs, V.R., 1982. Time preference and health: an exploratory study. In V. R. Fuchs, ed. *Economic Aspects of Health*. Chicago: University of Chicago Press.

Hagenaars, J.A. & McCutcheon, A.L., 2009. Applied Latent Class Analysis, Cambridge: Cambridge University Press.

Hirano, K. & Imbens, G.W., 2004. The Propensity Score with Continuous Treatments. In A. Gelman & X. L. Meng, eds. *Applied Bayesian Modeling and Causal Inference from Incomplete-Data Perspectives*. West Sussex, England: Wiley InterScience.

Jackson, M.O., 2011. An overview of social networks and economic applications. *Handbook of Social Economics*, 1, pp.511–585.

Kane, M.J., Poole, B.J., Tuholski, S.W. & Engle, R.W., 2006. Working memory capacity and the top-down control of visual search: Exploring the boundaries of “executive attention.” *Journal of Experimental Psychology-Learning Memory and Cognition*, 32(4), pp.749–777.

Kaufman, L. & Rousseeuw, P.J., 2009. Finding Groups in Data: An Introduction to Cluster Analysis, Hoboken, New Jersey: John Wiley & Sons.

Kluve, J., Schneider, H., Uhlendorff, A. & Zhao, Z., 2012. Evaluating continuous training programmes by using the generalized propensity score. *Journal of the Royal Statistical Society: Series A (Statistics in Society)*, 175(2), pp.587–617.

Kohler, U., Karlson, K.B. & Holm, A., 2011. Comparing coefficients of nested nonlinear probability models. *The Stata Journal*, 11(3), pp.420–38.

Lanza, S.T., Dziak, J.J., Huang, L., Wagner, A.T. & Collins, L.M., 2015. LCA Stata plugin users’ guide (Version 1.2), University Park: The Methodology Center, Penn State.

Manski, C.F., 1993. Identification of Endogenous Social Effects: The Reflection Problem. *Review of Economic Studies*, 60(3), pp.531–542.

Oberauer, K., Süß, H.-M., Schulze, R., Wilhelm, O. & Wittmann, W.W., 2000. Working memory capacity — facets of a cognitive ability construct. *Personality and Individual Differences*, 29, pp.1017–1045.

Perkins, J.M., Subramanian, S.V. & Christakis, N.A., 2014. Social networks and health: A systematic review of sociocentric network studies in low- and middle-income countries. *Social Science & Medicine*, 125, pp.60–78.

Picone, G., Sloan, F. & Taylor, Jr., D., 2004. Effects of Risk and Time Preference and Expected Longevity on Demand for Medical Tests. *Journal of Risk and Uncertainty*, 28(1), pp.39–53.

van der Pol, M., 2011. Health, education and time preference. *Health Economics*, 20(8), pp.917–929.

UNDP, 2014. Human Development Report 2014. Sustaining Human Progress: Reducing Vulnerabilities and Building Resilience. Available at: http://hdr.undp.org/en/content/human-development-report-2014.

University of Wisconsin Madison, 2015. Propensity Score Matching in Stata using teffects. Available at: https://www.ssc.wisc.edu/sscc/pubs/stata_psmatch.htm.

Vyas, S. & Kumaranayake, L., 2006. Constructing socio-economic status indices: How to use principal components analysis. *Health Policy and Planning*, 21, pp.459–468.

WHO, 2011. The Philippines Health System Review. *Health Systems in Transition*, 1(2).

1. Provinces are the main political and administrative entities in the Philippines. In 2015 there were 81 provinces which formed the 18 greater regions (17 administrative and 1 autonomous regions) of the country (http://www.nscb.gov.ph/activestats/psgc/listprov.asp). [↑](#footnote-ref-1)
2. Although this leads to a loss of information it helps improving the balancing of the sample, a necessary condition for the propensity score estimation. The main results remain largely consistent if we use continuous regional characteristics. However, in order to improve the balancing between the different treatment strata, we decided to restrict our analysis to the binary measures. [↑](#footnote-ref-2)
